# Multi-functionality of the few: current and past uses of wild plants for food and healing in Liubań region, Belarus

**DOI:** 10.1186/s13002-017-0139-x

**Published:** 2017-02-08

**Authors:** Renata Sõukand, Yanina Hrynevich, Iryna Vasilyeva, Julia Prakofjewa, Yuriy Vnukovich, Jury Paciupa, Aliaksei Hlushko, Yana Knureva, Yulia Litvinava, Siarhei Vyskvarka, Hanna Silivonchyk, Alena Paulava, Mare Kõiva, Raivo Kalle

**Affiliations:** 1 Estonian Literary Museum, Vanemuise 42, Tartu, 51003 Estonia; 2 The Center for Belarusian Culture, Language and Literature Research, Surhanava St., 1, Bldg. 2, Minsk, 220072 Belarus; 3 Valožynski district, v. Vialikaja Dajnava, Padhornaja st. 118, Minsk region, 222352 Belarus; 4 Liuban District Culture Center, Pieršamajskaja st., 30, Liuban, 223820 Belarus; 5 The Belarusian State University of Culture and Arts, Rabkoraŭskaja st. 17, Minsk, 220007 Belarus

**Keywords:** Belarus, Wild plants, Food plants, Medicinal plants, Food-medicine, Local knowledge, Ethnobotany, Ethnopharmacology, Ethnoveterinary, Liubań

## Abstract

**Background:**

This study examined the use of wild plants in the food, medicinal and veterinary areas within a small territory limited to one village council in the Liubań district of Belarus. The objectives of the research were to document the current and past uses of wild plants in this region for food and human/animal medication; to analyse the food, medicinal and veterinary areas in the context of wild plants; and to qualitatively compare the results with relevant publications concerning the wild food plants of Belarus.

**Methods:**

Fieldwork was carried out as a practical part of a development cooperation project in May 2016 in 11 villages of the Liubań district. One hundred thirty-four respondents were selected randomly. Information about local uses of wild plants was obtained via semi-structured interviews and the folk-history method. Interview records were digitalized and the data structured in Detailed Use Records (DUR), which were divided into food, medicinal and veterinary areas and then analysed to ascertain local perceptions.

**Results:**

A total of 2252 DUR of wild plants were recorded. Eighty-eight wild plant taxa belonging to 45 plant families were used across all three areas. Of these, 58 taxa were used in the food, 74 in the medicinal and 23 in the veterinary areas. A relatively high percentage of the taxa were used in both the food and medicinal areas (55%) and an even greater percentage in both the medicinal and veterinary areas (87%). Comparison with earlier research on wild food plants shows the considerable difference among seldom-mentioned taxa or uses, showing possible regional differences despite the homogenization of the population during the Soviet era.

**Conclusions:**

As the majority of taxa with overlapping uses belonged to the most utilized plants, there appears to be clear a tendency to use plants in several different areas once they are brought into the home. This may be due to the need to maximize the versatility of limited resources. While the number of wild taxa used is relatively high, the mean number of taxa used per person is quite low, which indicates the relatively minor importance of wild plants in the respective areas in the study region. The low importance of snacks signals that unintended contact with nature has been lost.

## Background

Life in present-day, highly literate Europe is rapidly changing. This affects local practices in the use of wild plants for food and healing in both people and animals, which is part of the Local Ecological Knowledge, crucial for sustaining human life on earth. Therefore, an increase of interest in documenting such knowledge and understanding the changes it undergoes is well justified. The classical studies by Etkin [1, 2] and Pieroni and Price [3], as well as more recent field examples in Europe [4–6], have demonstrated either a dual perception and function of food and medicinal uses and even their co-evolution, or a remarkable overlap in the nomenclature of species used simultaneously in the food and medicinal areas. Thus, the need to look at the uses of wild plants from both perspectives, food and medicine, is also warranted.

We can assume that the cultivation of plants brings them closer to people; the use of cultivated plants may increase as people have fewer encounters with the wild (for example [7]). Wild plants, in contrast, become more distant and less known, as a result of habitat loss, changes in the paths of human movement and the lessened need for wild supplies, as well as changes in cultural attitudes to wild foods or tastes [8, 9]. Simple logic will say that once the wild plant is brought home, it will be used for as many purposes as possible, for as long as the amounts gathered allow.

Luczaj et al. [10] referred to Belarus as *terra incognita* with respect to modern ethnobotany. Indeed, the majority of the information gathered about ethnomedicinal and food uses of wild plants originates from rather distant times. To date [10], remains the only comprehensive publication on the use of wild food plants in both modern and historical contexts in the territory of Belarus. The first records on traditional medicine in Belarus date to the late XVIII–early XIX centuries, which were included in descriptions of rituals and daily life of the local population. In general, the attention of researchers has been focused on the study of ways to treat the causes of diseases and their prevention. For example, the work titled “Description of the Barysaŭ district” by E. Tyszkiewicz provided a comprehensive list of plants (with local names) with their medicinal (also emic) purpose and usage both in the past and at the time of publication [11]. Throughout the XIX and early XX century a systematic collection and publication of ethnomedicinal field data was conducted for Belarus by numerous authors (for example YE. Romanov [12, 13], N. Nikiforovskiy [14–16], M. Federowski [17, 18] P. Shein [19, 20], V. Dobrovol'skiy [21] A. Bahdanovič [22], F. Wereńko [23], N. Yanchuk [24]). The main attention of these researchers was focused on documenting charms, information about the people involved in treatment and the variety of used remedies, including those involving plants. The unique information regarding the local names of plants of the Hrodna region and their medicinal properties are presented in the publication by E. Orzeszkowa [25]. Significant contribution to the systematization of the traditional concepts of Belarusians about the causes and origins of diseases and their treatment were made by Polish (K. Moszyński [26], Cz. Pietkiewicz [27], etc.) and Russian (D. Zelenin [28], G. Popov [29], etc.) scientists. During the Soviet era field research on traditional medicine passed out of scientific interest. As was common within other Soviet regions only a few “critical analyses” of ethnographic data from this period were published (Mińko [30, 31]), exploring methods of treatment, including the use of herbal medicine and magic. Recently, Belarusian folklorists have been actively studying the semiotic status and symbolic image of plants in traditional culture (I. Šved [32]), non-plant traditional medicine as part of the traditional culture (T. Valodzina [33–37]) and ethnographic classification of methods of treatments (U. Lobač [38] and U. Filipienka [39]). However, thus far no comprehensive and complex approach to the regional use of wild plants in Belarus has been published.

Therefore, in this study we decided to address one specific well-defined area of wild plants used for food and medicine pertaining to both humans and other animals within a little-studied territory. While classically such ethnobotanical fieldwork concentrates on food and medicinal uses of plants only, we choose to also include the veterinary area as domesticated animals are still kept in the researched region; and due to the widespread medical veterinary intervention this area is too small to be examined separately. The specific aims of this study are: 1) to document the current and past uses of wild plants in the Liubań district for food and human/animal medication; 2) to analyse the food, medicinal and veterinary areas in the context of wild plants; and 3) to qualitatively compare the results with relevant publications regarding the wild food plants of Belarus. We presume that wild plants are widely used in the region and remarkable overlap exists within the species used in both the food and medicinal areas.

## Data and methods

### Wild plants

In this article the concept of wild plants encompasses native and naturalized species not cultivated for food. The core of the selection is based on the internationally agreed upon ethnobotanical perception and refers primarily to plants growing without deliberate cultivation or those able to reproduce without human intervention [40–43]. It includes greenery trees (like *Syringa vulgaris*, *Aesculus hippocastanum*), but we excluded from the scope of this study all fruiting trees and shrubs cultivated for food purposes, even if they either run wild (like *Prunus cerasus* L. or *Ribes rubrum* L.) or have wild relatives (such as *Ribes nigrum* L. or *Malus* spp.), as a vast majority of people claimed that they collect fruits and other parts from the cultivated ones. As an exception we did include a taxon that was once cultivated, but now perceived somewhat as a nuisance (*Armoracia rusticana*). Other species that are cultivated for food purposes but tend to run wild (like *Anethum graveolens* L. or *Nigella sativa* L.) were excluded from this study.

### Region

**Belarus** is located in Central and Eastern Europe, bordered by Lithuania to the north-west, Latvia to the north, Russia to the northeast and the east, Ukraine to the south, and Poland to the west. Its territory is about 207.6 thousand sq. km. The climate of Belarus is moderately continental, transitional between maritime and continental. The climatic conditions are caused by the position of the country in the temperate latitudes of the western part of the East European Plain [44]. Belarus, more than other countries, was affected by the Chernobyl disaster, which caused the relocation of millions of people.

The whole territory of Belarus is part of the forest zone of Eastern Europe. Mixed forest is typical for Belarus, and from the north to the south there is a transition from boreal to nemoral forest types. Today, forests cover 37.8% of the total area, marshes 11.5%, grasslands 15.8% and bushes 1.9%. The vegetation varies with climate, soil type and topographic relief. Indigenous plants include about 100 species of trees and bushes as well as more than 1500 herbaceous plants [45]. The most common trees are conifers (pine, fir) and shrubs (juniper). The main deciduous species are birch, aspen, alder, as well as oaks and limes, and more rarely maple, hornbeam, ash, and poplar, among others. The most prominent shrubs are hazel, rowan, viburnum, buckthorn, raspberry, willow, vines, etc. The forests are also rich in mushrooms and berries, which includes strawberries, blueberries and cranberries in dry coniferous and mixed forests, and cranberries in swampy areas. Widespread meadow grasses include timothy, bluegrass, and various types of clover [45].

**Liubań** district is located in the southeast of the Minsk Region. The town of Liubań is the centre of the district which includes the township of Urečča and 125 rural settlements. The northern part of the district is located on the Central Biarezina plain, while the southern part is within the Prypiać Paliessie. In the central part of the district the Aresa River (a left-bank tributary of the Prypiać) flows from north to south. Most areas of the Paliessie region have been drained. About 33% of the area is covered by forests (coniferous and mixed deciduous forest, as well as birch, oak and alder that also grow there). The area is mostly agricultural and specializes in meat and milk cattle breeding, pig breeding and potato cultivation.

**Fig. 1:**
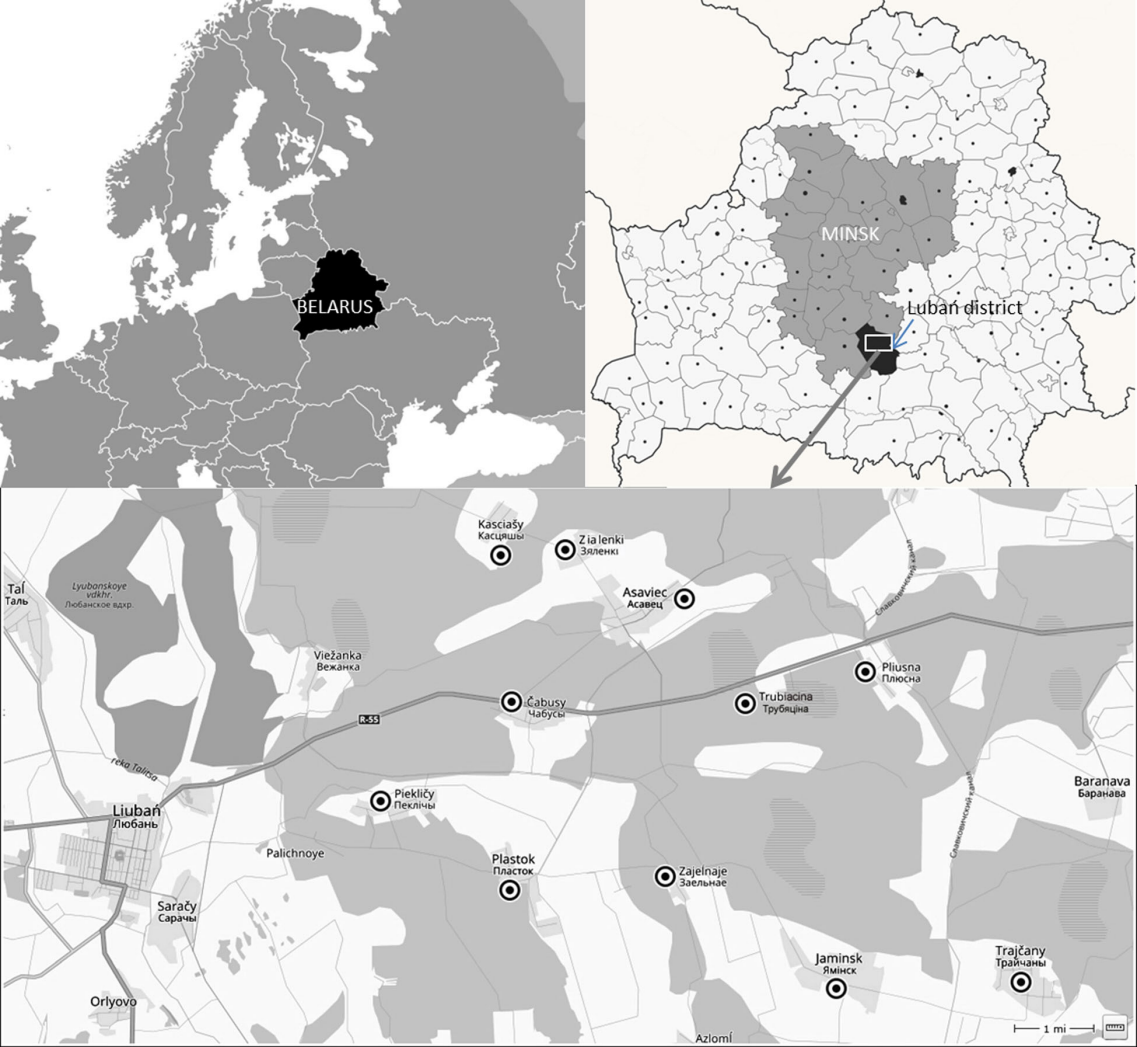
Region under investigation

Interviews were conducted in eleven villages, which are part of the **village council of Asaviec**, Liubań district (Fig. 1); for more information on the district see [46]. About 2450 inhabitants live in the 15 villages of the village council of Asaviec. The working-age population is dominated by men (men: 770; women: 533), while the reverse is true for the senior population (590 women and 221 men). Bigger villages, in particular, are home to numerous migrants from both Belarussian and Ukrainian parts of the Chernobyl radiation zone. In recent years, migrants from Donbas have also become common. They were first warmly received, but later locals became disappointed with their attitude: locals perceive that migrants consider life in the Asaviec region too hard and this makes them unsatisfied.

Orthodox Christianity is the religion of the vast majority of the rural population, although the Catholicism is also practiced. Unemployment officially does not exist; all working-age people are employed or have official exemptions for not working. Yet, some people admit that younger men temporally work in towns or in Russia to sustain their families, which is probably one reason for encountering so few young men. Retail shops are present in seven larger villages, where 100 to 900 people live; inhabitants of smaller villages are supplied by mobile shops visiting twice a week. Jaminsk has a hospital, and Plastok has a medical aid station served by a medical assistant/midwife. While some bigger villages have a better-developed water system, small villages have surface water wells, which can dry out in summer, and often one well is shared by several households. Toilets are mainly outside and the dominant washing place is the sauna. Houses are traditionally decorated inside and outside; as the wealth of the villagers allow, some renovation work (new windows, painting) is done, supported mainly by relatives living in towns.

Many villagers still keep domestic animals (pigs, goats, ducks, hens, cows and horses), but admit that their numbers have diminished during the last several years. Smaller villages are built mainly in a linear fashion, so that houses are situated on either side of the road with few cross streets, making the settlement long but compact. Almost every household has a bench in front, outside the fence and along the street, so that home owners can “socialize” with fellow villagers sitting on these benches. A lot of effort is put into maintaining vegetable gardens: the selection of fresh vegetables in shops is quite modest, and so all greens and vegetables are grown in relatively small garden plots close to houses. Flower gardens, which are considered the pride and joy of any good lady of the house, are more common in bigger villages, but even here they are confined to only a small part of the whole allotment.

### Data collection

The collection of data on the use of wild plants was part of a wider ethnobotanical and ethnomedicinal field study, conducted in the Liubań district in May 2016 as a practical part of a development cooperation project financed by the Estonian Foreign Ministry. Interviewees were approached on a random basis, in an attempt to get a cross-section of the local population. Yet, this was not simple, as mainly only retired people were available for discussions during workdays, whereas other adults were working or busy with household tasks (on the weekend). The majority of people in the study sample were collective farm workers, and only few of them had secondary or higher education (working as officials or in the sphere of culture). For the analysis we selected responses of all 134 people who were borneither in the villages where the interviews took place or elsewhere in the district of Li ubań (mainly from neighbouring villages belonging to the same village council) and now living in the village in which they were interviewed. Of these respondents, 87 (64.9%) were women and 47 (35.1%) were men. The fewer number of men is due to low representation of elderly men in the villages. The mean age of the interviewees was 63.3 years; the oldest was 92 years old and the youngest was 27 years old.

Interviews lasted from 0.5 to 2.5 h and were conducted either on the bench in front of the respondent’s house or in their homes, in which researchers were often invited (and then fed or offered (ritual) drinks), especially when it was raining. The folk history method (reconstruction of historical events through the memory of common people, sensu [47]) was employed: in addition to documenting current uses, we also asked interviewees to recall the uses of plants they had used themselves or observed their parents using in the past, especially during their childhood. In the semi-structured interviews we approached the subject through uses, not through specific plants. First, we inquired about the use of wild plants for food, naming specific food-use categories (soups, other hot dishes, salads, jams, desserts, put in bread, used for seasoning food and drinks, and snacks). As the interview proceeded, we asked about using plants and other means in healing (headache, cold, sore throat, heart problems, stomach ache, foot ache, wounds, etc.) both people and animals (when animals get ill) and also for other purposes related to health and wellbeing (prophylactics, being healthy, care of skin and hair, disinfection, good scent, etc.). We let the respondents elaborate on the subject. Where weather and the interviewee’s health or time permitted, walks in the gardens and surrounding meadows and forests were undertaken and voucher specimens collected. We also collected samples of dried plants available from the interviewees’ homes. Although it was late spring and most of the stores were already used up, we encountered a high variety of stored plants in many households. Interviews were voice-recorded upon permission of the interviewee, and field notes were also taken. The purpose of the study was explained to every person and prior informed consent was obtained from all interviewees. We followed the Code of Ethics of the International Society of Ethnobiology [48]. All interviews were subsequently transcribed. The voice-recorded interviews as well as their transcripts are stored at the The Center for Belarusian Culture, Language and Literature Research within the Archives of The Institute of Art, Ethnography and Folklore, named after K. Krapiva (AIAEF 23-16-2). Anonymized copies of interview transcripts are also stored at the Scientific Archive of the Estonian Folklore Institute (EFISA Valgevene2016) located in the Estonian Literary Museum.

Whenever possible plant voucher specimens were taken or plants identified on the basis of dried samples. As it was not yet the full vegetation season, some plants were identified on the basis of their vernacular name and a full description provided by the interviewee. In those cases when interviewees were treating a genus as one unit, the plant was identified at the genus level only, even if voucher specimens for some representatives of the genus were collected (for example Hypericum, Rosa, Crataegus). This practice was followed as there is no guarantee that interviewees, at some point in their lives, did not collect representatives of other species belonging to the same genus.

Collected voucher specimens were dried and identified with the help of Toomas Kukk (Curator of the Estonian University of Life Sciences herbaria); vouchers are deposited at the Estonian University of Life Sciences herbaria (TAA), assigned herbarium numbers within the range TAA0132555-0132710, and also bearing numbers LJUB001–152. Dried plant samples collected from respondents are deposited at the Scientific Archive of the Estonian Folklore Institute (EFISA Valgevene2016, bearing numbers LJUD001–85). Taxonomic identification, botanical nomenclature, and family assignments followed the Flora Europaea [49], The Plant List database [50], and the Angiosperm Phylogeny Group IV [51].

### Data analysis

Interview records were digitalized and entered into a Microsoft Excel spread sheet. To follow *emic* categories, information was structured in Detailed Use Records (DUR adopted from [52]), where interviewees (*i*) mention a specific use (*u*, e.g. emic disease category [cough, sore throat, heart disease, back pain, etc.], food category [snack, drink, condiment, soup, jam, etc.], emic veterinary treatment) of a plant part (*p*, e.g. fruits, leaves, aerial parts, flowers, etc.) prepared in a certain way (*w*, e.g. topical application of fresh plant, tea (plants macerated in hot water), decoction (plants boiled in water), tincture (plants macerated in alcohol–either applied or drunk), special preparation, etc.). Interviewee-defined emic categories were employed to ascertain local perceptions.

For every taxon, the number of Use Citations (UC–number of people who claimed the (specific) use of the plant during the interview) and the number of DUR were calculated for the sum of all uses and separately for food, medicinal and veterinary areas. UC were also calculated for general and emic categories.

Following the recommendation given in several recent publications [53, 54] and to illustrate the diversity of various uses, uses mentioned by only one person were also included. Informant Consensus Factor (FIC [55]) was calculated for every use area as well as for different use categories within each of the three areas. The FIC is calculated as follows: number of UC minus the number of species used, divided by the number of use citations minus one.

Finally, the reliability criterion [56] was also assessed. The influence of age of the interviewees on the number of used plants and DURs was assessed by calculating R^2^ in Microsoft Excel.

### Use areas

For the comparative analysis, all DURs were attributed to one of three use areas (hereafter areas):

- ***Food area***–indicates that the use was related to food consumption, including hot and cold meals, drinks (including alcoholic ones), fermented foods, condiments, occasional snacks, the making of recreational teas (e.g. herbal beverages prepared as infusions or decoctions and consumed in a food context without folk medical indications sensu [57]), food preservatives and food preparation accessories (e.g. tree leaves put under bread during baking).
- ***Medicinal area***–contains all uses related to the treatment or prevention of all diseases and illnesses that are locally known or diagnosed by a doctor, but also uses related to the perceived healthiness of a plant or product made from a plant, beauty procedures, insect repellent and the creation of a good atmosphere (contributing to wellbeing).
- ***Veterinary area***–covers all uses of wild plants related to the treatment of home animals or specially emphasized fodder, distinct from the general “hay, grains and potato” fodder.

For uses in the food area, qualitative comparison was made with data collected throughout Poland in 20th–21st centuries [10], while for the other two areas such a comparison was not possible due to the lack of recent data.

## Results

### Summary of the use of plants

Altogether, 88 wild plant taxa belonging to 45 plant families were used across all three areas (Table 1). Sixty-seven (76.1%) taxa met the reliability criterion, signifying that they were used by at least three people. We registered 2252 DUR of wild plants in total, with the food area dominating the variety of uses (54%, 1216 DUR), followed by uses related to the medicinal area (43%, 968 DUR) and the veterinary medicine and fodder area (3%, 68 DUR).

**Table 1:** Wild plants used in food, medicinal and veterinary areas

Family, taxa, voucher no.	Local names	Used parts	Mode of use	Emic use category	UC
Acoraceae					
*Acorus calamus* L.	aip, плюшнiк/air, pliušnik	Aerial parts	Placed in water	Disinfectant	1
		Roots	Ritual wearing	Evil eye	1
		Stem (lower, white part)	Fresh	Snack	1
Adoxaceae					
*Viburnum opulus* L., (LJUB036)	кaлiнa/kalina	Fruits	Dried	Recreational tea	1
			Dried on twigs	Snack	1
			Fermented	Heart diseases	2
				Hypertension	2
			Fresh	Cold	2
				Compote	1
				Dessert	1
				Fruit water	2
				Heart problems	1
				Hypertension	1
				Jam	5
				Kissel	1
				Taste additive to strong alcohol	6
				Snack	1
				Sore throat	1
				Syrup	1
				Wine	1
			Frozen	Snack	1
				Raw jam	1
			In sugar	Preserve	1
			Jam	Cold	1
			Raw jam	Cough	2
				Heart problems	1
			Tincture	Healthy	1
				Heart problems	1
				Hypertension	4
				Stomach ache	1
			Tea	Cough	1
		Flowers	Tea	Hypertension	1
		Leaves	Decoction	Sore throat	1
Amaranthaceae					
*Atriplex hortensis* L.	лeбядa кpacнaя/liebiada krasnaja	Aerial parts	Fresh	Soup	2
*Chenopodium album* L., (LJUB017)	лeбядa, лябядa/liebiada, liabiada	Aerial parts	Fresh	Salad	3
				Snack	3
				Soup	35
Amaryllidaceae					
*Allium ursinum* L., (LJUB002)	дзiкi чacнoк, чapaмшa, мeдвeжы лук/dziki časnok, čaramša, miedviežyj luk	Leaves	Dried	Condiment for meat	1
			Fresh	Salad	11
				Snack	5
				Soup	2
				Condiment	3
				Condiment for soup	1
				Vitamins	1
			Frozen	Snack	1
			Marinated	Condiment	2
		Roots	Fresh	Snack	1
Apiaceae					
*Aegopodium podagraria* L., (LJUD035)	cныць/snyć	Leaves	Fresh	Salad	1
*Carum carvi* L., (LJUB132)	кмiн, тмiн/kmin, tmin	Seeds	Dried	Recreational tea	1
				Condiment	3
				Condiment for bread	4
				Condiment for cheese	1
				Condiment for meat	1
				Condiment for sausages	1
Asparagaceae					
*Convallaria majalis* L.	лaндыш/landyš	Flowers	Tincture	Heart disease	2
*Maianthemum bifolium* (L.) F.W.Schmidt, (LJUB065)	мaйнiк/majnik	Fruits	Fresh	Snack	1
Asteraceae					
*Achillea millefolium* L., (LJUB118)	пaдбeл, тыcячaлicнiк/padbiel, tysiačalisnik	Aerial parts	Decoction	Heart problems	1
			Fresh	Fodder for turkey	1
			Tea	Bile neutralizer	1
				Gastritis	1
				Stomach ache	1
		Inflorescences	Tea	Panacea	1
				Stomach ache	2
				Women diseases	1
		Leaves	Decoction	Women diseases	1
			Topical application	Cuts	2
				Wounds	2
*Arctium tomentosum* Mill., (LJUB019)	лaпуx, лoпуx, вaўчкi, дзяды, дзядoўнiк, paпeйнiк/lapuch, lopuch, vaŭčki, dziady, dziadoŭnik, rapiejnik	Inflorescences	Decoction	Hair care	2
		Leaves	Fresh	Put under bread when baked	1
			Topical application	Back pain	1
				Bruises	1
				Foot ache	3
				Joint pain	9
				Knee ache	2
				Pain	1
				Painful place	3
				Rheumatic pains	2
				Tumour	1
				Wounds	1
		Roots	Decoction	Hair care	3
				Joint pain	1
			Fresh	Vitamins	1
			Fresh, greasing	Joint pain	4
			Tincture, topical	Foot ache	1
*Arnica montana* L.	apнiкa гopнaя/arnika hornaja	Whole plant	Tea	Heart problems	1
				Nerves	1
*Artemisia absinthium* L., (LJUB001)	пaлын (cepы), пaлыннiк/palyn (siery), palynnik	Aerial parts	Decoction	Appetizer for cows	1
				Diarrhoea in chicken	1
				Diarrhoea in cows	3
				Diarrhoea in pigs	5
				Rumination problems in cows	3
			Dried	Fodder for rabbits	2
			Fresh	Disinfectant for home animals	1
				Fodder for turkey	1
			Tincture, topical application	Joint pain	1
			Covered with	Preservative for potatoes	1
			Tea	Appetizer	2
				Diarrhoea	6
				Helminthic infection	1
*Artemisia vulgaris* L., (LJUB139)	чapнaбы’льнiк, чopны пaлын, быльнiк/čarnaby’ĺnik, čorny palyn, byĺnik	Aerial parts	Dried	Blood in urine in cows	1
				fodder for rabbits	1
			Fresh	Recreational tea	1
			Tea	Diarrhoea	2
*Bidens tripartita* L.	вaўчкi, чapaдa, чэpaдa/vaŭčki, čarada, čerada	Aerial part	Bath	Diathesis in children	6
				Calming	1
				Skin diseases	1
*Cyanus segetum* Hill, (LJUD023)	вaciлькi/vasiĺki	Aerial parts	Tea	Kidney diseases	1
		Inflorescences	Bath	Diathesis in children	1
			Compress	Eye problems	1
			Dried	Recreational tea	2
			Tea	Healthy	1
*Helichrysum arenarium* (L.) Moench, (LJUD007)	бяccмepтнiк (пяcчaны)/biassmiertnik (piasčany)	Aerial parts	Dried	Recreational tea	1
			Tea	Bile deficiency	1
				Gall stones	1
				Healthy	1
				Liver diseases	5
				Panacea	2
				Stomach ache	2
*Matricaria* spp. (Incl. *Matricaria chamomilla* L., (LJUD025) and *Matricaria suaveolens* Koch)	paмoнaк, paмaшкa/ramonak, ramaška	Aerial parts	Decoction	Calming	1
				Hair care	1
				Wounds	1
			Tea	Sore throat	1
				Stomach ache	1
		Inflorescences	Compress	Eye problems	2
			Decoction	Dandruff	1
				Hair care	1
				Heart problems	1
				Haemorrhoids	1
				Organism cleansing	2
				Women diseases	1
			Dried, eaten	Diarrhoea	1
*Tanacetum vulgare* L., (LJUB108)	цытвa’p, цытвa’pнaя пaлынь, пiжмa/cytva’r, cytva’rnaja palyń, pižma	Aerial parts	Fresh	Helminthic infection	1
			Tea	Diarrhoea	2
				Helminthic infection	1
				Stomach ache	2
		Inflorescences	Decoction	Helminthic infection	2
			Dried, added to fodder	Helminthic infection in cows	1
				Helminthic infection in pigs	1
				Strengthening of cows	1
				Strengthening of pigs	1
			Eaten fresh	Helminthic infection	1
				Stomach ache	1
			Tea	Diarrhoea	1
				Helminthic infection	1
		Leaves	Tea	Diarrhoea	1
				Helminthic infection	1
*Taraxacum officinale* (L.) Weber ex F.H.Wigg., (LJUB122)	дзьмуxaвeц, aдувaнчыкi/dźmuchaviec, aduvančyki	Aerial parts	Fresh	Fodder for pigs	1
			Tincture, topical application	Joint pain	1
		Inflorescences	Decoction	Joint pain	1
				Kidney diseases	1
			Fermented with sugar	Immune boosting	1
			Fresh	Cold	1
				Fodder for pigs	1
				Good for cows	1
				Good for horses	1
				Jam	10
			Fresh, topical application	Joint pain	2
			Jam	Bronchitis	1
				Cancer	1
				Cold	1
				Sore throat	1
				Tuberculosis	1
			Tincture	Stomach ache	1
			Tincture, topical application	Joint pain	1
			Syrup	Vitamins	1
		Leaves	Decoction	Hair care	1
			Fresh	Fodder for pigs	1
				Fodder for home animals	1
				Salad	7
			Tincture	Stomach ache	1
		Roots	Coffee substitute	Kidney stones	1
			Dried	Coffee substitute	1
				Recreational tea	1
			Fresh, eaten	Cancer	1
*Tussilago farfara* L., (LJUB023)	пaдбeл, мaцi-мaчыxa, мaць-мaчыxa/padbiel, maci-mačycha, mać-mačycha	Aerial parts	Topical application	Inflammation processes	1
		Inflorescences	Tea	Cold	1
				Panacea	1
		Leaves	Decoction	Healthy	1
				Headache	1
				Sore throat	1
			Tea	Cold	1
				Cough	5
				Expectorant	1
				Panacea	1
			Topical application	Headache	1
				Wounds	1
Betulaceae					
*Alnus* spp.	aльxa, aлeшнiк/aĺcha, aliešnik	Buds	Bath	Foot hatching	1
		Cone-like fruits	Decoction	Diarrhoea in cows	1
				Diarrhoea in pigs	1
				Diarrhoea	4
				Stomach ache	1
*Betula* spp., (incl. *Betula pendula* Roth (LJUB081))	бяpoзa, бepёeзa/biaroza, berioza	Buds	Dried	Taste additive to strong alcohol	1
			Tincture	Diarrhoea	2
				Epilepsy	2
				Gastric ulcer	1
				Gastritis	1
				Healthy	1
				Kidney diseases	3
				Panacea	3
				Sore throat	2
				Stomach ache	3
				Strengthening of organism	1
				Wounds	3
			Ritually fermented, bath	Epilepsy	1
			Tea	Cold	2
				Diuretic	1
				Heart problems	1
				Panacea	2
				Sore throat	1
		Catkins	Tincture	Diarrhoea	1
				Gastric ulcer	1
				Gastritis	1
				Panacea	1
				Stomach ache	1
			Tincture, topical application	Joint pain	1
			Macerated in water	Diarrhoea in calves	1
			Tea	Cold	1
		Leaves	Bath	Allergy	1
			Dried	Recreational tea	2
			Tea	Cold	1
				Heart problems	1
				Panacea	1
				Stomach ache	1
			Topical application	Foot ache	1
		Resin	Topical application	Wounds	2
		Sap	Fermented	Kvass	36
			Fresh	Drink	30
				Instead of water	2
				Diuretic	1
				Increase volume of breast milk	1
			Processed	Drink	16
				Kidney diseases	1
		Twigs	Bath	Prophylactics	1
			Whisked in sauna	Back pain	1
				Healthy	5
				Promotes bloodstream	1
				Prophylactics	10
*Corylus avellana* L.	apэx (ляcны), фундук, apэшынa/arech (liasny), funduk, arešyna	Seeds	Dried	Snack	3
			Fresh	Snack	5
Boraginaceae					
*Pulmonaria* spp.	мeдунiцa/miedunica	Flowers	Fresh	Snack	1
*Symphytum officinale* L.	aкoпнiк/akopnik	Leaves	Topical application	Bruises	1
		Roots	Tincture	Adhesion of bones	1
Brassicaceae					
*Armoracia rusticana* Gaertn. et al., (LJUB093)	xpэн/chren	Leaves	Fresh	Preservative for preserves	1
				Condiment	3
				Condiment for lactofermented cucumbers	8
				Condiment for preserves	1
			Topical application	Back pain	1
				Joint pain	2
		Roots	Dried	Condiment for meat	1
			Fresh	Appetizer	1
				Disinfectant	1
				Healthy	1
				Taste additive to strong alcohol	1
				Salad	3
				Snack	2
				Condiment	13
				Condiment for beet juice	1
				Condiment for lactofermented cucumbers	1
				Condiment for meat	3
				Condiment for pork fat	1
			Macerated in beer	Diabetes	1
*Capsella bursa-pastoris* (L.) Medik., (LJUB098)	пacтуш’я cумкa, cумaчкa, cушaнкa/pastušja sumka, sumačka, sušanka	Aerial parts	Decoction	Haemostatic	1
			Tea	Diarrhoea	1
				Stomach ache	1
				Urinary bladder	1
		Seeds	Dried	Bread additive	1
Cannabaceae					
*Humulus lupulus* L., (LJUD027)	xмeль/chmieĺ	Cones	Decoction	Insomnia	1
			Dried	Beer	1
			Fresh	Bread additive	1
				Taste additive to strong alcohol	1
		Leaves	Dried	Beer	1
Caprifoliaceae					
*Valeriana officinalis* L.	вaлepьян, вaляp’янкa/valieŕjan, valiarjanka	Aerial parts	Dried	Recreational tea	1
			Tincture	Healthy	1
			Tea	Headache	1
				Heart problems	2
				Sedative	1
		Roots	Decoction	Heart problems	2
				Joint pain	1
				Organism cleansing	1
			Tincture	Calming	1
				Heart problems	2
			Tea	Calming	1
				Headache	1
Caryophyllaceae					
*Stellaria media* (L.) Vill., (LJUB024)	мaкpэц, мaкpiцa, звecчaткa/makrec, makrica, zviesčatka	Aerial parts	Decoction	Cancer	1
			Fresh	Salad	2
			Tincture, topical application	Rheumatic pains	1
			Fresh, topical application	Foot sores	1
				Headache	1
				Rheumatic pains	2
Cupressaceae					
*Juniperus communis* L.	ядлoвeц, мaжжaвeльнiк/jadloviec, mažžavieĺnik	Galbules	Tincture	Back pain	1
		Leaves	Decoction	Bronchitis	1
		Pollen	Powder	Skin diseases	1
		Twigs	Tea	Cold	1
				Cold in domesticated animals	1
			Whisked in sauna	Healthy	2
				Prophylactics	2
Dryopteridaceae					
*Dryopteris carthusiana* (Vill.) H.P. Fuchs, (LJUB083)	пaпapaтнiк/paparatnik	Leaves	Bath	Rejuvenating	1
Equisetaceae					
*Equisetum arvense* L., (LJUB120)	xвoшч пaлявы/chvošč paliavy	Aerial parts	Decoction	Bacterial diseases in bees	1
			Tea	Diuretic	1
				Healthy	1
				Kidney diseases	1
Ericaceae					
*Arctostaphylos uva-ursi* (L.) Spreng.	тaлaкнянкa/talaknianka	Aerial parts	Tea	Kidney diseases	1
*Calluna vulgaris* (L.) Hull, (LJUD079)	вepecк/vieriesk	Flowers	Dried	Recreational tea	1
*Chimaphila umbellata* (L.) Nutt., (LJUB146)	cтaнaўнiк/stanaŭnik	Aerial parts	Tea	Stomach ache	1
			Tincture	Stomach ache	1
*Ledum palustre* L., (LJUD052)	бaгoн, бaгoўнiк, буячнiк/bahon, bahoŭnik, bujačnik	Aerial parts	Decoction	Diarrhoea in calves	1
				Diarrhoea in cows	1
				Bronchitis	1
				Diarrhoea	1
			Dried	Recreational tea	1
			Smoked	Tick prevention	1
			Tea	Cough	3
				Nerves	1
*Vaccinium myrtillus* L., (LJUB026)	чapнiкa, чapнiчнiк, iвaнaвыя ягaды, чopныя ягaды, ягoднiк, ягaды (лecныe)/čarnicy, čarnika, čarničnik, ivanavyja jahady, čornyja jahady, jahodnik, jahady (liesnyje)	Aerial parts	Dried	Recreational tea	6
			Tincture	Improve vision	1
			Tea	Diabetes	1
				Panacea	1
			Topical application	Rejuvenating	1
		Fruits	Compote	Eye problems	1
				Improve vision	1
			Decoction	Cold	1
			Dried	Compote	8
				Constipation	1
				Diabetes	1
				Diarrhoea	12
				Eye problems	4
				Healthy	1
				Taste additive to strong alcohol	1
				Recreational tea	4
				Snack	8
				Stomach ache	7
				Teething pain in children	1
				Dessert	1
			Dried, ritual	Stomach ache	1
			Fresh	Compote	26
				Dessert	3
				Diarrhoea	2
				Eye problems	5
				Fruit water	1
				Healthy	1
				Hypotension	1
				Improve vision	3
				Dessert	1
				Jam	55
				Juice	1
				Kissel	4
				Taste additive to strong alcohol	6
				Snack	31
				Stomach ache	2
				Syrup	2
			Frozen	Raw jam	2
				Pies	1
				Snack	6
			Jam	Eye problems	1
				Stomach ache	3
			Processed	Pies	1
				Dessert	1
			Sap dropped into eye	Eye problems	1
			Tea	Diarrhoea	1
*Vaccinium oxycoccos* L.	жуpaвiны, клюквa/žuraviny, kliukva	Fruits	Dried	Recreational tea	1
			fermented	Wine	1
			Fresh	Cold	2
				Compote	4
				Dessert	1
				Fruit water	2
				Hypertension	2
				Jam	11
				Juice	2
				Kissel	2
				Taste additive to strong alcohol	2
				Snack	22
				Condiment for lactofermented cucumbers	1
				Condiment for sauerkraut	9
			fresh applied in ear	CO-intoxication	1
			Frozen	Raw jam	1
				Snacks	5
			Fruit water	Cold	1
				Hangover	1
				Hypertension	1
			Tincture	Hypertension	1
			Preserved in water	Snack	1
			Tea	Gastric ulcer	1
				Panacea	1
			Under water	Snack	1
*Vaccinium uliginosum* L., (LJUD076)	буякi, гaлубiкa/bujaki, halubika	Aerial parts	Tea	Healthy	1
		Fruits	Compote	Anaemia	1
				Bad blood	1
			Dried	Cold	1
				Compote	3
				Taste additive to strong alcohol	1
				Recreational tea	2
				Snack	1
			Fresh	Compote	8
				Jam	19
				Kissel	1
				Taste additive to strong alcohol	2
				Snack	21
			Frozen	Snack	4
				Raw jam	1
			Tea	Diuretic	2
*Vaccinium vitis-idaea* L., (LJUB008)	бpуcнiцы, бpуcнiкa, бpуcнiчнiк/brusnicy, brusnika, brusničnik	Aerial parts	Dried	Recreational tea	9
			Fresh	Recreational tea	1
			Frozen	Frozen	2
			Tea	Diuretic	1
				Fever	1
				Healthy	1
				Heart problems	2
				Kidney diseases	1
				Panacea	1
				Stomach ache	1
		Fruits	Cooked	Tonic	1
			Dried	Compote	3
				Snack	1
			Fresh	Compote	6
				Dessert	1
				Fruit water	1
				Jam	35
				Kissel	2
				Liver diseases	1
				Taste additive to strong alcohol	1
				Snack	14
				Condiment for sauerkraut	1
				Syrup	1
				Wine	1
			Frozen	Snack	4
			Fruit water	Kidney diseases	1
				Sore throat	1
			Processed	Pies	2
			Refrigerated	Raw jam	1
			Tea	Healthy	1
				Organism cleaning	1
		Leaves	Dried	Recreational tea	5
			Tea	Cold	1
				Constipation	1
				Diuretic	1
				Hypertension	1
				Kidney diseases	5
				Panacea	1
				Urinating problems	1
				Women diseases	1
Fagaceae					
*Populus tremula* L., (LJUD054)	aciнa, тoпaль/asina, topaĺ	Bark	Compress	Joint pain	1
			Decoction	Panacea	1
			Tincture	Panacea	1
			Pulverized, eaten	Kidney diseases	2
			Tea	Immune boosting	1
		Buds	Tincture	Joint pain	1
		Leaves	Topical application	Wounds	1
		Sap	Fermented	Preservative for birch sap	1
		Sticks	Put into bed	Aching legs	1
		Twigs	Fresh	Additive to compote	1
			Topical application	Convulses	1
*Quercus robur* L., (LJUB119)	дуб/dub	Acorns	Dried, grounded	Bread additive	3
				Appetizer for cows	1
			Fresh	Bread additive	1
			Milled into flour	Fodder for home animals	1
		Bark	Bath	Diathesis in children	1
				Foot hatching	1
			Decoction	Bruises	1
				Diarrhoea	3
				Diarrhoea in cows	2
				Diarrhoea in pigs	1
				Gingival diseases	1
				Panacea	1
				Periodontitis	1
			Dried	Coffee substitute	1
				Taste additive to strong alcohol	1
				Condiment	1
				Condiment for fermented birch sap	1
			Fresh	Snack	1
				Condiment for lactofermented cucumbers	2
			Macerated in hot water	Fodder for calves	1
		Leaves	Fresh	Preservative for preserve	1
				Put under bread when baked	10
				Condiment for kvass	1
				Condiment for lactofermented cucumbers	5
				Under bread	2
			Inhalation	Hypertension	1
		Malformation of the leave	Juice, topical application	Wounds	1
			Topical application	Warts	1
		Twigs	Bath	Prophylactics	1
			Fresh	Preservative for kvass	3
				Condiment for fermented birch sap	1
				Condiment for lactofermented cucumbers	1
			Whisked in sauna	Back pain	1
				Healthy	5
				Promotes bloodstream	1
				Prophylactics	5
Hydrangeaceae					
*Philadelphus coronarius* L.	жacмiн/žasmin	Leaves	Dried	Recreational tea	1
Hypericaceae					
*Hypericum* spp., incl. *Hypericum perforatum* (LJUB095)	cвeтaяннiк, звepaбoй/svietajannik, zvieraboj	Aerial parts	Bath	Body cleansing	1
			Decoction	Diarrhoea in cows	1
				Heart problems	1
				Rumination problems in goats	1
				Scabies	1
			Dried	Taste additive to strong alcohol	2
				Recreational tea	12
				Condiment	1
			Fresh	Stomach ache	1
			Tincture, drunk	For women to be strong against men	1
				Healthy	3
				Kidney diseases	1
			Smoked	When piglets do not go to their mother	1
			Tea	Cold	7
				Cough	3
				Diarrhoea	2
				Fright	1
				Heart problems	4
				Liver diseases	1
				Panacea	8
				Pancreas	1
				Sore throat	1
				Stomach problems	1
				Stomach ache	4
				Tonus support	1
				Varix	2
Lamiaceae					
*Comarum palustre* L., (LJUB063)	caбeльнiк/sabieĺnik	Roots	Tincture, topical application	Joint pain	3
*Origanum vulgare* L., (LJUB043)	мaцяpдушкa, душыцa, мaцяpдушкa/maciarduška, dušyca	Aerial parts	Dried	Recreational tea	2
			Tea	Calming	1
				Cold	1
				Stomach problems	2
				Women diseases	1
*Thymus serpyllum* L. (s.l.), (LJUB037)	вepac, чaбapoк, чaбapэц, чaбop/vieras, čabarok, čabarec, čabor	Aerial parts	Dried	Recreational tea	8
			Tea	Cold	3
				Cough	5
				Hypertension	1
Leguminosae					
*Trifolium* spp., (LJUD053)	кaнюшынa, клeвep/kaniušyna, klievier	Aerial parts	Fresh	Fodder for rabbits	1
				Fodder for pigs	1
		Flowers	Tea	Heart diseases	2
Melanthiaceae					
*Veratrum lobelianum* Berhn.	чaмяpыцa/čamiaryca	Aerial parts	Decoction	Rumination problems in cows	1
			Fresh	Rumination problems in cows	2
Oleaceae					
*Syringa vulgaris* L., (LJUB071)	бэз (бeлы), cipэнь (бeлaя)/bez (biely), sireń (bielaja)	Buds	Tincture	Joint pain	1
		Flowers	Decoction	Joint pain	1
			Dried	Recreational tea	1
			Fresh	Snack for luck	1
				Snack	1
			Tincture	Healthy	1
				Stomach ache	1
			Tincture, topical application	Rheumatic pains	6
				Joint pain	5
				Foot ache	1
				Back pain	1
		Leaves	Fresh, topical application	Cuts	1
Onagraceae					
*Epilobium angustifolium* L.	iвaн-чaй/ivan-čaj	Aerial parts	Dried	Recreational tea	3
			Tea	Vitamins	1
			Whisked in sauna	Healthy	1
		Flowers	Decoction	Headache	1
				Hypertension	1
			Tea	Healthy	1
				Hypertension	1
				Kidney diseases	1
		Leaves	Dried	Recreational tea	1
Orobanchaceae					
*Lathraea squamaria* L.	пятpoў кpэcт/piatroŭ krest	Roots	Fresh	Vitamins for cows	1
Oxalidaceae					
*Oxalis acetosella* L., (LJUB109)	зaчый шчaвeль, кicлiцa, зaячaя кaпуcтa, вepaбeйкaў шчaвeль/začyj ščavieĺ, kislica, zajačaja kapusta, vierabiejkaŭ ščavieĺ	Leaves	Fresh	Salad	1
				Snack	9
Papaveraceae					
*Chelidonium majus* L., (LJUB140)	paннiк, цындaлeй, чыcтaцeл, чicтaцeл/rannik, cyndaliej, čystaciel, čistaciel	Aerial parts	Bath	Allergy	1
				Body cleansing	3
				Diathesis in children	3
				Eczema	1
				Healthy	1
			Decoction	Diarrhoea in cows and pigs	1
				Beauty procedure	1
				Chickenpox	1
				Wounds	1
			Kvass	Organism cleaning	1
			Tincture	Stomach problems	1
			Tincture, topical application	Rheumatic pains	1
			Stewed	Scabies	1
			Tea	Body cleansing	1
				Liver diseases	1
		Leaves	Fresh, snacked	Healthy	1
			Topical application	Eczema	1
				Burns	2
				Cuts	3
				Toothache	1
				Wounds	4
		Sap	Diluted juice, lavation	Gingival bleeding	2
			Intake	Gastric ulcer	1
				Allergy	1
				Prophylactics	1
			Topical application	Haemostatic	2
				Skin diseases	2
				Warts	9
				Wounds	9
*Papaver* spp.	мaк, мaк-вiдук, дзiкi мaк, мaк-caмaceй/mak, mak-viduk, dziki mak, mak-samasiej	Seeds	Decoction	Calming	1
			Dried	Pies	1
				Soporific	1
				Condiment	1
				Condiment for bread	1
				Condiment for pancakes	1
			Fresh	Helminthic infection	1
				Pies	1
				Snack	1
				Soporific	1
Pinaceae					
*Picea abies* (L.) H.Karst., (LJUB121)	eлкa/jelka	Needles	Foot bath	Joint pain	1
		Twigs	Whisked in sauna	Healthy	1
		Young cones	Fresh	Jam	1
			Tincture	Healthy	1
			Tea	Cold	1
*Pinus sylvestris* L., (LJUB082)	cacнa, xвoя/sasna, chvoja	Pollen	Whisked in sauna	Healthy	1
		Resin	Topical application	Wounds	1
				Wounds in cows	1
		Shoots	Fresh	Jam	3
			Processed	Recreational tea	1
			Tea	Liver diseases	1
				Lung diseases	1
				Stomach ache	1
		Twigs	Whisked in sauna	Healthy	1
				Prophylactics	1
		Young cones	Decoction	Cold	1
			Fresh	Jam	3
				Snack	1
			Jam	Bronchitis	1
				Cold	3
				Cough	1
				Kidney diseases	1
				Liver diseases	1
				Sore throat	1
				Stomach ache	1
			Tincture, topical application	Joint pain	1
				Rheumatic pains	1
			Processed	Recreational tea	2
			Tea	Bronchitis	1
				Cold	2
				Cough	1
				Immune boosting	2
				Inflammation processes	2
				Stomach ache	1
				Strengthening of organism	2
				Thyroid glands	1
				Tuberculosis	1
Plantaginaceae					
*Plantago major* L., (LJUB106)	пaдapoжнiк/padarožnik	Leaves	Fresh	Salad	1
				Stomach ache	1
			Juice pressed	Gastric ulcer	1
			Tea	Diarrhoea	1
				Heart problems	2
				Low acidity	1
				Kidney diseases	1
			Topical application	Abscess	3
				Burns	1
				Cuts	4
				Cuts in domesticated animals	1
				Gingival wounds	1
				Headache	3
				Haemostatic	1
				Wounds	32
Poaceae					
*Elymus repens* (L.) Gould, (LJUB031)	пыpaй, пыpeй, пыpэй/pyraj, pyriej, pyrej	Leaves	Fresh	Salad	1
			Topical application	Joint pain	1
		Roots	Compress	Eye problems	1
			Decoction	Joint pain	1
			Fresh	Soup	1
				Condiment for salad	1
			Tincture, topical application	Joint pain	1
*Phleum* spp.	цiмaфeeўкa, тpaвa/cimafiejeŭka, trava	Aerial parts	Fresh, fodder	To increase cow milk production	1
Polemoniaceae					
*Polemonium caeruleum* L.	ciню́гa гaлубaя/siniúha halubaja	Roots	Tincture	Epilepsy	1
Polygonaceae					
*Polygonum aviculare* L., (LJUB042)	cпapыш, тpaўкa-муpaўкa/sparyš, traŭka-muraŭka	Aerial parts	Tea	Diarrhoea	1
				Kidney diseases	1
			Topical application	Headache	1
*Rumex* spp. (incl. *Rumex acetosa* L., (LJUB151))	шчaўe, шчaвeль, вepaб’iны, шчaвeль дзiкi, шчaвeль ляcны, шчaвep/ščaŭje, ščavieĺ, ščavieĺ vierabjiny, ščavieĺ dziki, ščavieĺ liasny, ščavier	Leaves	Fresh	Preserve	1
				Snack	14
				Soup	61
				Stomach problems	1
			Frozen	Soup	1
			Processed	Soup	14
*Rumex longifolius* DC., (LJUB136)	кoнcкi шчaвeль/konski ščavieĺ	Seeds	Tea	Diarrhoea	1
Rhamnaceae					
*Frangula alnus* Mill., (LJUB015)	кpушынa/krušyna	Bark	Tea	Diarrhoea	1
		Fruits	Fresh	Constipation	1
				Dysentery	2
Rosaceae					
*Crataegus* spp., (LJUB085)	бaяpышнiк/bajaryšnik	Fruits	Decoction	Heart problems	1
			Dried	Condiment, processed birch sap	1
			Tincture	Heart diseases	1
				Hypertension	1
			Tea	Heart problems	3
				Hypertension	1
		Leaves	Tea	Heart problems	2
		Twigs with fruits	Tincture	Heart problems	1
*Fragaria vesca* L., (LJUB048)	cунiцы, зeмлянiкa, зeмлянiчнiк/sunicy, ziemlianika, ziemlianičnik	Aerial parts	Decoction	Heart problems	2
				Kidney diseases	1
			Dried	Recreational tea	1
			Fresh	Recreational tea	1
			Tea	Liver diseases	1
				Pneumonia	1
		Fruits	Dried	Compote	2
				Liver diseases	2
				Snack	1
			Fresh	Compote	5
				Dessert	2
				Jam	17
				Snack	16
			Frozen	Dessert	1
				Snack	2
			Tea	Cold	1
		Leaves	Dried	Recreational tea	5
			Tea	Healthy	1
*Potentilla erecta* (L.) Raeusch., (LJUB047)	дзiвaciл, кaлгaн, дpaўлянкa, лaпчaткa/dzivasil, kalhan, draŭlianka, lapčatka	Roots	Decoction	Antitoxic	1
				Stomach ache	1
			Dried	Taste additive to strong alcohol	4
			Tincture	Antimicrobic	1
				Gastric ulcer	2
				Healthy	5
				Heart problems	1
				Inflammation	1
				Kidney diseases	3
				Sore throat	1
				Stomach problems	1
				Stomach ache	12
				Thyroid glands	1
			Tincture, topical application	Burns	1
				Cuts	1
				Rotten wounds	1
			Tea	Diarrhoea	1
				Gastritis	1
				Panacea	1
				Stomach ache	4
*Prunus padus* L., (LJUB084)	чapoмxa/čaromcha	Flowers	Tincture, topical application	Joint pain	1
				Rheumatic pains	1
		Fruits	Compote	Diarrhoea	1
			Fresh	Diarrhoea	2
				Dysentery	2
				Snack	2
			Tincture	Diarrhoea	1
		Twigs	Placed in water	Disinfectant	1
*Pyrus pyraster* (L.) Burgsd., (LJUB050)	гpушы дзiкiя, дзiчкa/hrušy dzikija, dzička	Fruits	Decoction	Diarrhoea	5
				Diarrhoea in cows	1
			Compote	Cold	1
				Diarrhoea	1
				Stomach ache	1
			Dried	Cold	1
				Compote	4
				Diarrhoea	4
				Healthy	1
				Recreational tea	1
				Snack	2
				Stomach ache	2
			Fermented	Kvass	2
			Fresh	Jam	1
				Snack	3
				Fodder for pigs	1
*Rosa* spp., (LJUD001)	шыпшынa, шыпoўнiк, шыпoвнiк/šypšyna, šypoŭnik, šypovnik	Fruits	Decoction	Heart problems	1
			Dried	Recreational tea	8
				Condiment for processed birch sap	1
			Fresh	Compote	2
				Jam	3
			Tincture	Stomach ache	1
			Tea	Cold	2
				Healthy	1
				Heart diseases	1
				Hypertension	1
				Immune boosting	3
				Inflammation processes	2
				Kidney diseases	3
				Liver diseases	2
				Panacea	2
*Rubus caesius* L., (LJUB003)	aжыны, eжaвiкa (лecнaя)/ažyny, ježavika (liesnaja)	Fruits	Fresh	Compote	2
				Dessert	2
				Jam	14
				Kissel	1
				Taste additive to strong alcohol	1
				Recreational tea	1
				Snack	8
			Frozen	Hypertension	1
				Liver diseases	1
				Snack	2
			Processed	Pies	1
		Leaves	Dried	Recreational tea	3
		Twigs	Dried	Recreational tea	1
		Twigs with fruits	Dried	Recreational tea	1
*Rubus idaeus* L., (LJUB044)	мaлiнa (ляcнaя), мaлiннiк/malina (liasnaja), malinnik	Aerial parts	Dried	Recreational tea	7
			Fresh	Snack	1
			Tea	Cold	1
				Cough	1
				Healthy	1
				Sore throat	2
		Fruits	Compote	Cough	1
			Dried	Cold	1
				Compote	1
				Cough	1
				Recreational tea	2
				Snack	1
				Condiment	1
			Fermented with sugar	Prophylactics	1
			Fresh	Cold	2
				Compote	11
				Heart problems	1
				Jam	34
				Juice	1
				Kissel	1
				Taste additive to strong alcohol	3
				Snack	16
			Frozen	Cold	1
				Dessert	1
				Frozen	5
				Jam	1
				Raw jam	2
			Fruit water	Cold	1
			Jam	Cold	7
				Cough	2
				Fever	1
				Sore throat	4
			Tincture	Soporific	1
				Stomach ache	1
			Processed	Pies	1
			Tea	Cold	4
				Cough	1
				Sore throat	2
		Leaves	Dried	Recreational tea	9
			Tea	Bronchitis	1
				Cold	6
				Cough	3
				Sore throat	1
				Strengthening of organism	1
		Twigs	Dried	Recreational tea	7
			Fresh	Recreational tea	1
			Tea	Cold	4
				Cough	2
				Rhinitis	1
		Twigs with fruits	Dried	Recreational tea	1
		Twigs with leaves	Dried	Recreational tea	1
			Tea	Cold	3
				Constipation	1
				Cough	1
				Pneumonia	1
*Rubus saxatilis* L.	кaмянiкa, кacцянiкa/kamianika, kascianika	Fruits	Fresh	Snack	2
*Sorbus aucuparia* L., (LJUB075)	apaбiнa, кpacнaя paбiнa/arabina, krasnaja rabina	Flowers	Tea	Cold	1
		Fruits	Decoction	Bone strengthening	1
			Dried	Hypotension	1
				Recreational tea	1
			Fermented	Wine	1
			Fermented with sugar	Prophylactics	1
			Fresh	Cold	1
				Jam	2
				Snack	1
				Syrup	1
			Frozen on twigs	CO-intoxication	1
			Raw jam	Cough	2
			Tincture	Healthy	2
		Leaves	Tea	Hypotension	1
Salicaceae					
*Salix* spp.	вяpбa cвeчaнaя, iвa/viarba sviečanaja, iva	Seeds	Massage	Prophylactics	1
		Shoots	Tea	Cold	1
Santalaceae					
*Viscum album* L., (LJUB074)	aмялa/amiala	Leaves	Fresh	Organism cleansing	1
Sapindaceae					
*Acer platanoides* L., (LJUB131)	клён/klion	Leaves	Fresh	Put under bread	4
			Dried, from twigs blessed on Pentecost, topical application	Wounds	2
		Sap	Boiled into syrup	Fodder for bees in spring	1
			Fermented	Kvass	2
			Fresh	Drink	10
				Snack	3
			Processed	Drink	1
		Twigs	Presence in house	Aroma therapy	1
			Whisked in sauna	Healthy	1
				Prophylactics	2
*Aesculus hippocastanum* L., (LJUB111)	кaштaн/kaštan	Flowers	Decoction	Joint pain	1
			Tincture, topical application	Foot ache	2
				Joint pain	3
				Rheumatic pains	2
		Fruits	Tincture, topical application	Rheumatic pains	1
				Joint pain	2
		Pericarp	Decoction	Varix	2
Scrophulariaceae					
*Verbascum thapsus* L., (LJUB062)	дзiвaciл/dzivasil	Aerial parts	Decoction	Panacea	1
			Tincture	Liver diseases	1
			Tincture, topical application	Joint pain	1
			Tea	Lung diseases	1
		Flowers	Tea	Stomach problems	1
		Leaves	Tincture	Stomach ache	1
			Tea	Stomach ache	1
		Roots	Tea	Healthy	1
Solanaceae					
*Datura stramonium* L.	шaлянeц, дуpмaн/šalianiec, durman	Fruits	Tincture	Diarrhoea	1
Tiliaceae					
*Tilia cordata* Mill., (LJUB089)	лiпa/lipa	Buds	Decoction	Cold	2
		Cambium	Boiled in milk	Abscess	1
		Flowers	Dried	Taste additive to strong alcohol	1
				Recreational tea	32
			Fresh	Taste additive to strong alcohol	1
				Rhinitis	1
			Tea	Cold	17
				Cough	7
				Diarrhoea	1
				Healthy	3
				Headache	1
				Hypertension	1
				Sore throat	1
		Leaves	Dried	Recreational tea	1
			Tea	Healthy	1
		Twigs	Whisked in sauna	Cold	1
				Healthy	1
Typhaceae					
*Typha* spp.	paгoз, кaмыш/rahoz, kamyš	Fuzz	Topical application	Burns	3
Urticaceae					
*Urtica dioica* L., (LJUB087)	кpaпiвa/krapiva	Aerial parts	Decoction	Fodder for cows	1
				Hair care	6
				Strengthening of piglets	1
				Thickening of blood	1
				Washing hair	1
			Dried	Fodder for cows	1
			Fresh	Salad	3
				Snack	1
				Soup	17
				Fodder for cows	1
				Strengthening of pigs	3
			Processed	Soup	1
			Topical application	Back pain	1
		Leaves	Decoction	Hair care	3
			Dried	Condiment for soup	1
			Fresh	Bread additive	1
				Cutlets	1
				Heart problems	1
				Organism cleansing	1
				Salad	4
				Snack	4
				Soup	19
				Condiment for salad	1
			Scaled, topical application	Rheumatic pains	1
			Tea	Diabetes	2
				Urinary bladder	1
				Vesical cleansing	1
				Women diseases	1
			Washing	Hair care	1
		Seeds	Fresh	Soup	2
*Urtica urens* L.	кpaпiвa пякучкa, кpaпiвa жыжкa/krapiva piakučka, krapiva žyžka	Aerial parts	Decoction	Hair care	1
			Fresh	Fodder for chicks	1
				Preservation of food	2
				Soup	4
				Fodder for turkey	1
Violaceae					
*Viola arvensis* Murray, (LJUB009)	бpaткi, iвaн-ды-мap’я, бpaткi/bratki, ivan-dy-marja	Aerial parts	Tea	Fever	1
		Flowers	Bath	Diathesis in children	1
			Dried	Recreational tea	1
			Topical application	Earache	1
		Leaves	Tea	Cold	1
			Topical application	Wounds	1
		Roots	Tea	Panacea	1

Local plant names are provided in both Cyrillic and Latin script

The four plant families with the highest number of taxa and DUR were Asteraceae (12 taxa/206 DUR), Rosaceae (10 taxa/420 DUR), Ericaceae (8 taxa/501 DUR), and Betulaceae (3 taxa/170 DUR). Among the ten most diversely used taxa, the vast majority were fruit bearing shrubs (four *Vaccinium* genera and *Rubus idaeus*), trees (*Betula* spp., *Tilia cordata* and *Quercus robur*) and herbaceous taxa (*Rumex* spp. and *Urtica dioica*).

**Fig. 2:**
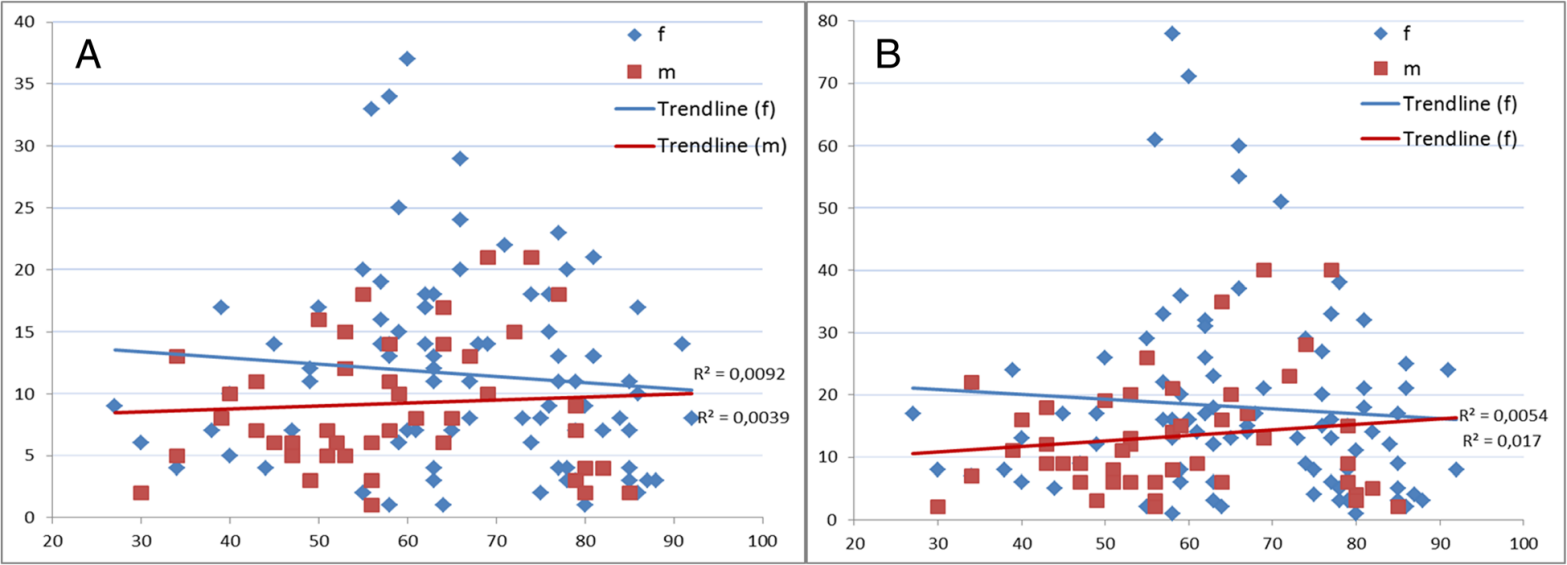
Distribution of the number of taxa used (**a**) and DUR (**b**) according to the age and sex of interviewees

The two most knowledgeable interviewees provided 39 and 36 taxa and 73 and 81 uses, respectively. The two least knowledgeable respondents provided only one use. The mean number of named taxa was 11.6 and mean number of DUR was 16.8. Although visually a small group of middle-aged women stand out as having more diverse knowledge, there was no statistically significant difference in the influence of age or sex of the respondents on either the number of used plants or the diversity of uses (Fig. 2).

### Food area

In total, 58 taxa belonging to 31 families were used for food. Of these, only 19 taxa were mentioned by more than 10% of the interviewees, while the uses of 26 taxa were mentioned by less than three people; the reliability criterion was met by 55.2% (32) of the taxa. The most represented families were Rosaceae (10 taxa), Asteraceae (6 taxa) and Ericaceae (6 taxa). The families with the most varied food uses were Ericaceae (391 DUR), Rosaceae (240 DUR), Betulaceae (95 DUR), Polygonaceae (91 DUR), and Urticaceae (61 DUR). The most diversely used food taxa overlap greatly with the top taxa overall and only *Fragaria vesca* and *Chenopodium album* replaced *Quercus robur* and *Tilia cordata*, which dominate the list of medicinal plants.

Among the taxa used by at least three respondents, four were used solely for food : *Chenopodium album*, *Oxalis acetosella*, *Carum carvi* and *Corylus avellana*, but only the first of these was used by a large number of interviewees (41).

Among the 124 respondents who claimed to have used wild plants for food, the two most knowledgeable used 18 taxa, numbering 30 and 29 DUR respectively. However, there were seven people who mentioned only one use of wild plants for food. The mean number of used taxa was 7.3 and the mean number of DUR was 9.08.

**Fig. 3:**
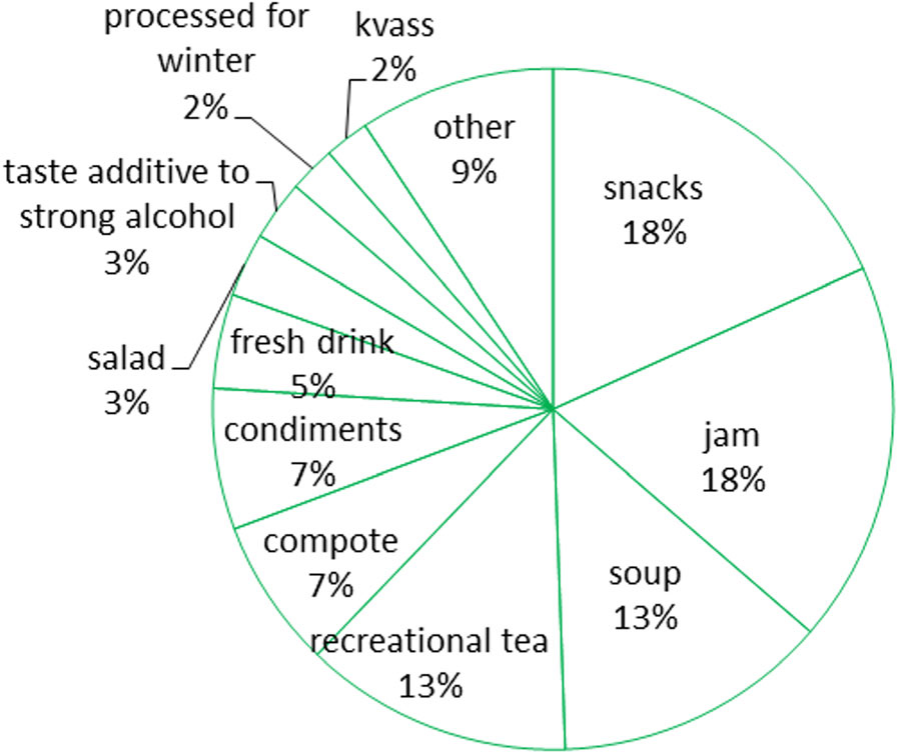
Division of DUR in the food area

All the DUR in the food area were distributed between 52 emic food categories, which can be attributed to 23 general food categories (Table 2). However, more than three-quarters of all DUR were distributed between six general food categories (Fig. 3).

**Table 2:** General categories in food area, their compostition and Use Citations

General food category	Emic food category	UC
Bread additive	Bread additive	7
Compote (87)	Compote	86
	Additive to compote	1
Condiments for food (82)	Condiment for soup	2
	Condiment for sausages	1
	Condiment for sauerkraut	10
	Condiment for salad	2
	Condiment for processed birch sap	2
	Condiment for preserves	1
	Condiment for pork fat	1
	Condiment for pancakes	1
	Condiment for meat	6
	Condiment for lactofermented cucumbers	18
	Condiment for fermented birch sap	3
	Condiment for chees	1
	Condiment for bread	5
	Condiment for beet juice	1
	Condiment	28
Dessert	Dessert	15
Fresh and processed drink	Fresh and processed drink	45
Fruit water	Fruit water	6
Jam (222)	Raw jam	8
	Jam	214
Juice	Juice	13
Kissel	Kissel	12
*Kvass*	Kvass	40
Other (6)	Instead of water	1
	Cutlets	1
	Coffee	2
	Beer	2
Pies	Pies	8
Preservative (9)	Preservative for preserve	2
	Preservative for potatoes	1
	Preservative for kvass	3
	Preservative for birch sap	1
	Preservation of food	2
Processed for winter (27)	Snacks	24
	Preserve	2
	Drink	1
Put under bread	Put under bread	17
Recreational tea	Recreational tea	156
Salad	Salad	37
Snack (223)	Snack	222
	Ritual snack	1
Soup	Soup	160
Syrup	Syrup	5
Taste additive to strong alcohol	Taste additive to strong alcohol	35
Wine	Wine	4

The Informant Consensus Factor for the whole food area was 0.94 (910 use citations for 58 taxa). Only one general food category had a relatively high FIC while having only two utilized taxa: **fresh and processed drinks** (FIC = 0.98), represented by tree sap. The category of **kvass** (FIC = 0.95), which is also mainly tree sap based, contains three taxa; the same number of taxa was in the category of leaves that are put under bread (FIC = 0.88). Some of the dominate categories with a relatively wide variety of used taxa attained high FIC values; however, the majority of those contain some highly dominate taxa and about twice the number of taxa used by only a few people. Foremost in this group was the **soup** category (FIC = 0.96), where among seven taxa three were highly prevalent (*Rumex acetosa*, *Chenopodium album* and *Urtica dioica*), followed by **compote** (FIC = 0.88), dominated by two forest berries (*Vaccinium uliginosum* and *Vaccinium myrtillus*), and **condiments** (FIC = 0.84) dominated by *Armoracia rusticana, Quercus robur, Carum carvi* and *Vaccinium oxycoccos*.

The most homogenous general category was **jam**, which possessed one of the highest FIC values (0.94). Eight of its 14 taxa, all but one of them (*Taraxacum officinale*) forest berries, were used by over 10 interviewees. Quite equivocal is the category of **recreational tea**, which contained the highest number of taxa (29) and a relatively high FIC (0.81): five taxa (*Tilia cordata*, *Rubus idaeus*, *Vaccinium vitis-idaea*, *Hypericum* spp., *Vaccinium myrtillus*) were mentioned by 10 or more interviewees, while 18 taxa were mentioned by only one or two respondents. A similar pattern was observed for **snacks** (FIC = 0.87), which is highly dominated by fruits (the most numerous of them being *Vaccinium myrtillus*, *Vaccinium oxycoccos*, *Vaccinium uliginosum*, *Fragaria vesca*, and *Rubus idaeus*), but also contains a wide variety of occasionally mentioned taxa.

### Comparison with data known about the 20th–21st centuries in food area

The number of taxa (67) outlined in [10] is greater than that recorded in this study (58). However, as we excluded from the list fruiting trees and shrubs that are mainly collected from cultivation, the difference in numbers is below 10%. The difference in taxa used is remarkable for snacks, which are well represented in [10], but rather rare in the present study. Several other uses such as *Matricaria* spp. and *Artemisia absinthium* listed in [10] as recreational teas, were used in this region, solely as medicine. In addition, *Berberis vulgaris*, for example, was not used, but we also did not encounter any shrubs in the region. For some taxa, the difference was on the level of different species from the same genus (*Viola*).

More than ten taxa were added to the known modern uses of recreational teas in Belarus by the results of this study. Of these, the reliability criterion was met by seven taxa (in order of popularity): *Vaccinium vitis-idaea*, *Hypericum* spp., *Vaccinium myrtillus*, *Fragaria vesca*, *Rubus caesius*, *Epilobium angustifolium*, and *Pinus sylvestris*. We also documented the use of *Prunus padus* and *Maianthemum bifolium* as snacks, which have been observed elsewhere (for example in Estonia [58, 59]), but not in Belarus.

We recorded some uses which were absent in recent data, but present in historical sources, such as various uses of the sap of *Acer platanoides*, or the use of *Papaver* spp. in bread and sweets. Also, the use of *Chenopodium album* in soups is solely referred to as “former” in [10], but this study showed that it is still used, although less intensely than in the past.

### Medicinal area

Medicinal use was reported for 74 taxa belonging to 38 families. Of these, only 16 taxa were mentioned by more than 10% of the interviewees, while 19 taxa were mentioned by less than three people; the reliability criterion was met by 74.3% (55) of the taxa. The most diversely used families were Rosaceae (9 taxa/178 DUR), Asteraceae (12 taxa/151 DUR), Ericaceae (7 taxa/108 DUR), Betulaceae (2 taxa/72 DUR), Papaveraceae (2 taxa/61 DUR) and Plantaginaceae (1 taxa/52 DUR). The ten most popular taxa in this area differed from the overall top list considerably more than did wild food plants: *Betula* spp., *Rubus idaeus*, *Vaccinium myrtillus*, *Chelidonium majus*, *Plantago major*, *Hypericum* spp., *Potentilla erecta*, *Tilia cordata*, *Arctium tomentosum*, and *Quercus robur*.

The medicinal area contained 15 taxa not used in the food area, which were named by more than three interviewees. Only one of these (*Tanacetum vulgare*), however, was mentioned by more than 10% of interviewees (14). The majority of the other 14 taxa (*Tussilago farfara, Aesculus hippocastanum, Achillea millefolium, Matricaria spp., Juniperus communis, Bidens tripartita, Verbascum thapsus, Alnus* spp*., Frangula alnus, Trifolium* spp.*, Equisetum arvense, Comarum palustre, Typha* spp*., Polygonum aviculare*) are common and well-known medicinal plants in the former Soviet states.

Among the 113 respondents who claimed to have used wild plants in the medicinal area, the three most knowledgeable used 29, 27 and 26 taxa, numbering 42, 34 and 54 DUR, respectively. Eleven people named only one use of wild plants in the medicinal area. The mean number of used taxa was 6.2 and the mean number of DUR was 8.6.

**Fig. 4:**
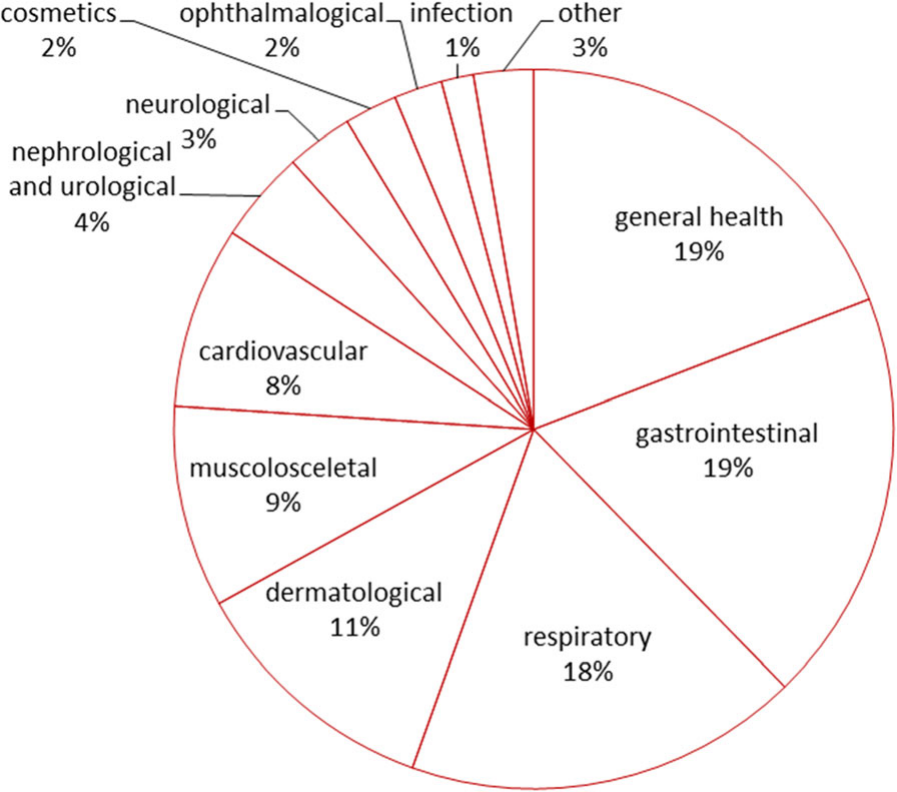
Division of DUR in the medicinal area

All the DUR in the medicinal area were distributed between 122 emic categories, which can be attributed to 16 general categories related to health and wellbeing (Table 3). However, 85% of all DUR are distributed between six general categories (Fig. 4).

**Table 3:** General categories in medicinal area, included emic categories and Use Citations

General categories in medicinal area (sum of DUR)	Emic disease categories	UC
Cardiovascular (82)	Vesical cleaning	1
	Varix	4
	Thickening of blood	1
	Promotes bloodstream	2
	Hypotension	3
	Hypertension	24
	Haemostatic	4
	Heart problem	34
	Heart disease	9
	Bad blood	1
	Anaemia	1
Cosmetics (23)	Rejuvenating	2
	Hair care	19
	Dandruff	1
	Beauty procedure	1
Culture bound (3)	Fright	1
	For women to be strong against men	1
	Evil eye	1
Dermatological (98)	Wounds	62
	Warts	10
	Tumour	1
	Skin diseases	4
	Scabies	1
	Rotten wounds	1
	Inflammation processes	1
	Foot hatching	3
	Eczema	2
	Cuts	11
	Burns	7
	Allergy	1
	Abscess	4
Endocrinological (8)	Thyroid glands	2
	Pancreas	1
	Diabetes	5
Gastrointestinal (180)	Stomach ache	69
	Stomach problems	7
	Low acidity	1
	Liver diseases	17
	Haemorrhoids	1
	Gastritis	4
	Gastric ulcer	7
	Gall stones	1
	Dysentery	4
	Diarrhoea	63
	Constipation	4
	Bile neutralizer	1
	Bile deficiency	1
General health (182)	Vitamins	4
	Washing hair	1
	Tonus support	1
	Tonic	1
	Tick prevention	1
	Strengthening of organism	4
	Prophylactics	26
	Panacea	32
	Pain	1
	Organism cleansing	7
	Inflammation processes	5
	Immune boosting	7
	Healthy	55
	Hangover	1
	Foot ache	1
	Fever	3
	Disinfectant	3
	Diathesis in children	12
	CO-intoxication	2
	Cancer	3
	Bone strengthening	1
	Body cleansing	5
	Aroma therapy	1
	Appetizer	3
	Allergy	2
Gynaecological (7)	Women diseases	6
	To increase milk production in woman	1
Infection (14)	Tuberculosis	2
	Scabies	1
	Helminthic infection	9
	Chickenpox	1
	Anti-microbic	1
Musculoskeletal (91)	Joint pain	50
	Rheumatic pains	17
	Knee ache	2
	Adhesion of bones	1
	Foot ache	7
	Bruises	3
	Painful place	3
	Back pain	7
	Aching legs	1
Nephrological and urological (40)	Urinating problems	1
	Urinary bladder	2
	Kidney stones	1
	Kidney diseases	29
	Diuretic	7
Neurological (29)	Soporific	3
	Sedative	1
	Nerves	2
	Insomnia	1
	Headache	11
	Epilepsy	4
	Convulses	1
	Calming	6
Ophthalmological (21)	Improve vision	5
	Eye problems	16
Oral and dental (7)	Toothache	1
	Teething pain in children	1
	Periodontitis	1
	Gingival diseases	1
	Gingival bleeding	2
	Gingival wound	1
Outer influence (1)	Antitoxic	1
Respiratory (172)	Sore throat	22
	Rhinitis	2
	Pneumonia	2
	Lung diseases	2
	Expectorant	1
	Earache	1
	Cough	42
	Cold	94
	Bronchitis	6

The Informant Consensus Factor for the whole medicinal area was 0.92 (698 use citations for 74 taxa). All general categories in the medicinal area had lower FIC values and only seven general categories had a FIC over 0.7.

The category of **respiratory** illnesses with a FIC value of 0.84 (26 taxa) was clearly dominated by two taxa: *Rubus idaeus* and *Tilia cordata,* both used primarily to treat cold, cough and sore throat; two more taxa (*Hypericum* spp. and *Pinus sylvestris*) were named by more than 10 people and used mainly against cold. Among the rest there were three taxa used in a certain mode that met the reliability criterion: tea made from the aerial parts of *Thymus serpyllum* and *Ledum palustre* or leaves of *Tussilago farfara* to alleviate cough; 54% of taxa in the respiratory category were used by less than three respondents.

The category of **ophthalmological** diseases had a similarly high FIC (0.84), but one of the smallest use citation numbers (20). This category also had a very small number of used taxa (four), dominated by one taxon: *Vaccinium myrtillus*, which is considered good for eye problems and believed to improve vision.

**Dermatological diseases** (FIC = 0.81) contained 21 taxa, two of which were dominant: *Plantago major* with the well-known topical application of fresh leaves on wounds, burns, cuts and abscesses, and *Chelidonium majus* with a wide variety of applications and modes of use, including the topical application of sap on wounds (said to be *welded*) and warts, as well as treating other skin diseases and injuries (like eczema, cuts, burns) by applying leaves, and a decoction of the aerial parts for external application to treat eczema and also washing wounds. As for the other taxa uses in the dermatology category, the reliability criterion was met only by the use of a tincture made from the buds of *Betula* spp. for cleansing wounds and the topical application of the fuzz of *Typha* spp. to treat burns. All of the remaining taxa used in the dermatological category (including the internationally well-known haemostatic *Achillea millefolium*) were mentioned by less than five people and did not meet the reliability criterion for a specific application mode.

**The gastrointestinal category** (FIC = 0.77) contained 37 taxa (almost half of them were mentioned by less than three respondents). The fruits of two of the three most dominant taxa, *Vaccinium myrtillus* and *Pyrus pyraster,* were utilized in a wide variety of use modes mainly to treat diarrhoea. A tincture made from the roots of *Potentilla erecta* was primarily used to alleviate stomach ache. Of the remaining taxa-use combinations, only a few met the reliability criterion: seven taxa used against three emic disease categories. The most common of these was diarrhoea, which was treated with a tea or decoction of the aerial parts of *Artemisia absinthium* and *Tanacetum vulgare*, the cone-like fruits of *Alnus* spp., and the bark of *Quercus robur* as well as a tincture made from the buds or catkins of *Betula* spp. The other emic disease categories that met the reliability criterion were stomach ache, which was treated with tea made from the aerial parts of *Hypericum* spp. and a tincture of *Betula* spp., and the use of a tea made from *Helichrysum arenarium* to treat liver disease.

The category of **general health** (FIC = 0.75) contained the highest number of taxa in one general category (46). It was dominated by four taxa: *Betula* spp. (used mainly as a prophylactic and healthy whisking in sauna, but also a tincture made from the buds as a panacea), *Hypericum* spp. (tea as a panacea), *Quercus robur* L. (wellbeing and prophylactic whisking in sauna) and *Chelidonium majus* (decoction used as a bath against diathesis in children and for body cleansing). Of the other combinations of taxa and uses, the reliability criterion was met by four taxa used on three occasions: an alcoholic infusion of the roots of *Potentilla erecta* and a tea made from the inflorescences of *Tilia cordata* were considered healthy, a bath of the decoction of *Bidens tripartita* was used against diathesis in children and a tea made from the fruits of *Rosa* spp. for boosting the immune system.

**The musculoskeletal** category (FIC = 0.74) contained 22 taxa, three of which were used by more than ten people. The fresh leaves of *Arctium tomentosum* were mainly topically applied to alleviate joint and foot pain. A tincture of *Syringa vulgaris* was applied on rheumatic and joint pains and a tincture made from the inflorescences or fruits of *Aesculus hippocastanum* was used for the same purpose. Of the remaining taxa only two more met the reliability criterion: *Taraxacum officinale* was used in a variety of modes, and a tincture of *Comarum palustre* topically applied to alleviate joint pain.

Within other general categories, only a few uses met the reliability criterion. In the **cardiovascular category** this included a tincture made from the fruits of *Viburnum opulus* used against hypertension, as well as a tea or decoction of the aerial parts or roots of *Valeriana officinalis* and a tea made from the fruits of *Crataegus* spp. or the areal parts of *Hypericum* spp. to treat heart problems. In the **nephrological and urological** category the leaves of *Vaccinium vitis-idaea* or the fruits of *Rosa* spp. and a tincture made from the buds of *Betula* spp. or roots of *Potentilla erecta* were used to treat kidney diseases. The **cosmetics** category was represented mainly by the decoction of the aerial parts or leaves of *Urtica dioica* and the decoction of the roots and flowers of *Arctium tomentosum* for washing hair. The only use that met the reliability criterion in the **neurological** category was the topical application of the leaves of *Plantago major* to alleviate headache.

### Veterinary area

Twenty-three taxa belonging to 17 families were used for ethnoveterinary purposes and as fodder. Only three families were represented by more than one taxon: Asteraceae (five taxa), Betulaceae (two taxa), and Urticaceae (2 taxa); whereas the most diversely used taxa were Asteraceae (30 DUR), Urticaceae (9 DUR) and Fagaceae (6 DUR). All the uses within the veterinary area were mentioned by less than 10% of the respondents, and only six taxa (*Artemisia absinthium*, *Urtica dioica*, *Taraxacum officinale*, *Quercus robur*, *Veratrum lobelianum*, and *Hypericum* spp.) were used by more than two people; therefore the reliability criterion was met by only a small percentage of taxa (26%). Three taxa were uniquely used in this area: *Lathraea squamaria* and *Phleum* spp. were both named by one interviewee and *Veratrum lobelianum* was mentioned by three persons. Seven taxa (*Achillea millefolium*, *Alnus* spp., *Chelidonium majus*, *Equisetum arvense*, *Juniperus communis*, *Tanacetum vulgare* and *Trifolium* spp.) overlap with taxa used for healing humans but not with those used for human food; all but the last were used by only one person.

Among the 34 people who claimed to have used wild plants in the veterinary area, the two most knowledgeable used six and three taxa, numbering eight and six DUR, respectively. Seventeen people (50% of all respondents who mentioned the veterinary use of wild plants) named only a single use of wild plants. The mean number of used taxa was 1.6 and the mean number of DUR was 2.0.

**Fig. 5:**
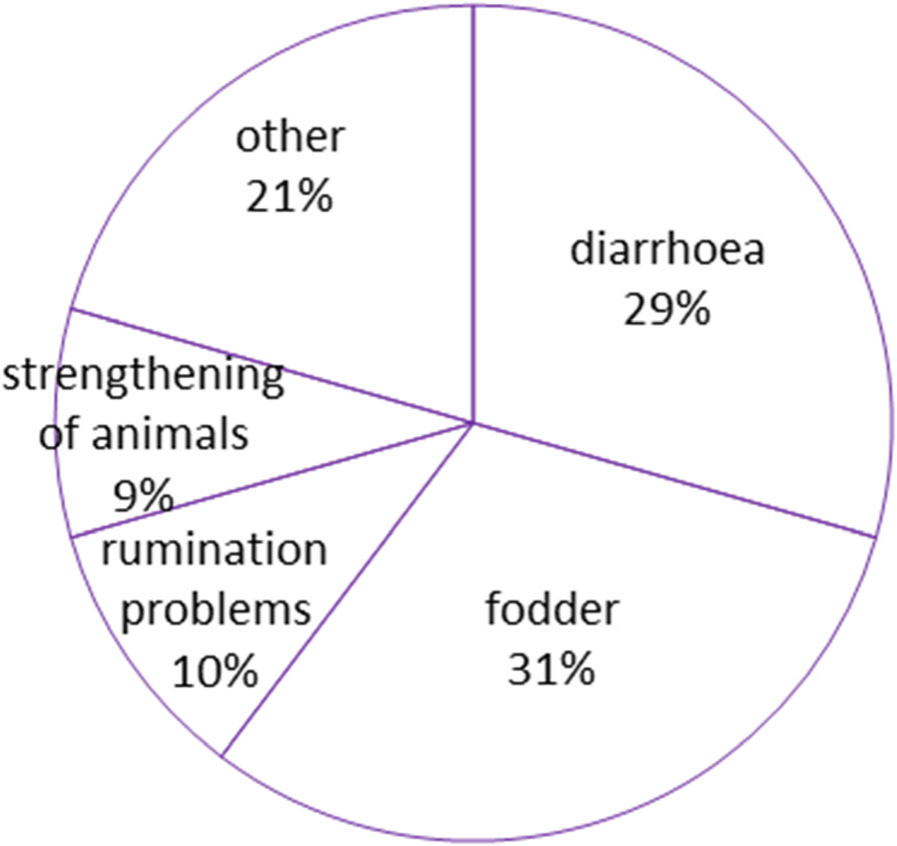
Division of DUR in the veterinary area

All the DUR in the ethnoveterinary area were distributed between 33 emic categories, which were attributed to three main general categories related to the healing of animals and to one category for fodder; 21% of DUR belonged to diverse emic categories of which not a single use met the reliability criterion (Table 4, Fig. 5).

**Table 4:** General categories in veterinary area, included emic veterinary categories and Use Citations

Food category	Emic veterinary categories	UC
Diarrhoea (20)	Diarrhoea in calves	2
	Diarrhoea in chicken	1
	Diarrhoea in cows	10
	Diarrhoea in pigs	8
Fodder (21)	Fodder for bees in spring	1
	Fodder for calves	1
	Fodder for chicks	1
	Fodder for cows	3
	Fodder for cows, to increase milk production	1
	Fodder for home animals	2
	Fodder for pigs	5
	Fodder for rabbits	4
	Fodder for turkey	3
Other (14)	Appetizer for cows	2
	Bacterial diseases in bees	1
	Blood in urine in cows	1
	Cold in domesticated animals	1
	Cuts in domesticated animals	1
	Disinfectant for home animals	1
	Good for cows	1
	Good for horses	1
	Helminthic infection in cows	1
	Helminthic infection in pigs	1
	When piglets do not go to their mother	1
	Vitamins for cows	1
	Wounds in cows	1
Rumination problems (7)	Rumination problems in cows	6
	Rumination problems in goats	1
Strengthening of animals (6)	Strengthening of cows	1
	Strengthening of piglets	1
	Strengthening of pigs	4

The Informant Consensus Factor for the whole veterinary area was low 0.67 (68 use citations for 23 taxa). However, only one general veterinary category (rumination problems) had a FIC equivalent to the FIC of the area, with two taxa (*Artemisia absinthium* and *Veratrum lobelianum*) meeting the reliability criterion for this specific use. One other category, **gastrointestinal**, had FIC = 0.63 (eight taxa all used against diarrhoea) with two taxa meeting reliability criterion: *Artemisia absinthium* for pigs and cows (and chickens) and *Quercus robur* for pigs and cows. The remaining categories had relatively low Informant Consensus Factors (0.25–0.5). Among the specific taxa meeting the reliability criterion within a specific emic category were dry *Urtica dioica* aerial parts added to fodder to strengthen pigs and fodder for cows, *Taraxacum officinale* used as fodder for pigs, *Artemisia absinthium* as fodder for rabbits and turkey.

### Overlap between areas

**Fig. 6:**
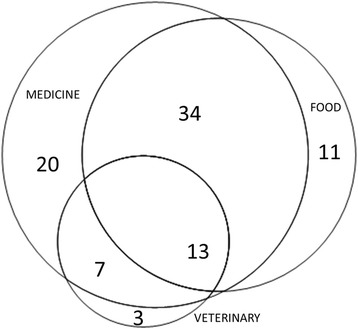
Overlap between taxa used in food, medicinal and veterinary areas

Figure 6 illustrates the overlap of taxa used in all three areas. Forty-seven taxa were used in both the food and medicinal areas, which equates to an overlap of 55.3%. The overlap between medicinal and veterinary areas is only 27% (20 taxa), but given that almost 87% of all taxa used in the veterinary area are also used in the medicinal area indicates that there is considerable overlap here as well (Fig. 6).

**Fig. 7:**
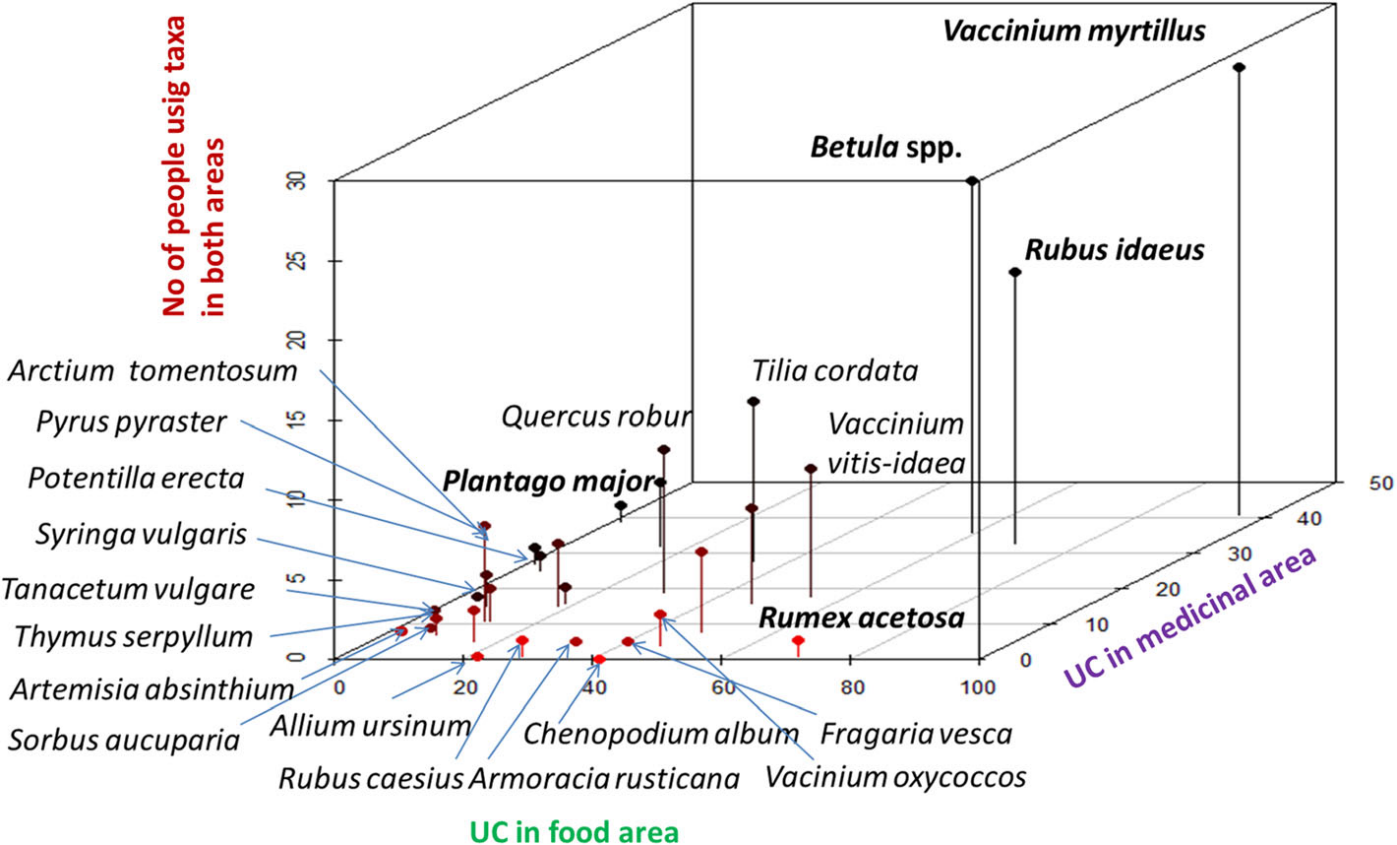
3D scatter plot of the overlapping use of 30 plants mentioned by at least 10% of the interviewees in the food and medicinal areas; designed in R [65]

**Fig. 8:**
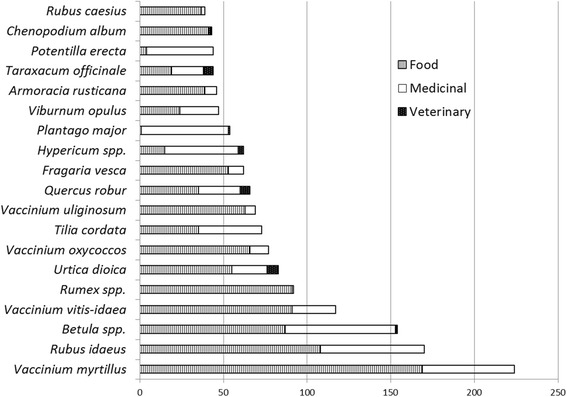
The twenty most intensively used taxa and the distribution of DUR between the three areas

Thirty wild plant taxa had more widespread use, that is they were used by at least 14 (approximately 10%) of the interviewees (Table 5). A comparison of the plants in the food and medicinal areas shows that the more widespread the use, the more there were people using one plant in both areas (most notably *Vaccinium myrtillus*, *Betula* spp. and *Rubus idaeus*), while there were specific taxa represented in both areas but predominantly used in only one, such as *Rumex* spp. for food and *Plantago major* as medicine (Fig. 7). The prevalence of plants used in the food area is clearly seen among the 20 taxa with the highest number of DUR, as medicinal use prevails in only three taxa (*Plantago major*, *Potentilla erecta*, *Hypericum* spp.) (Fig. 8).

**Table 5:** Taxa used by at least 10% of the interveiwees

Taxa	U *n* = 134	DUR	F_U *n* = 124	F_DUR	M_U *n* = 113	M_DUR	Overlap F_U/M_U	V_U *n* = 34	V_DUR
*Vaccinium myrtillus*	108	224	95	169	41	55	28		
*Rubus idaeus*	85	170	69	108	33	62	17		
*Betula* spp.	73	154	59	87	36	66	22	1	1
*Rumex* spp.	71	92	71	91	1	1	1		
*Vaccinium vitis-idaea*	64	117	54	91	18	26	8		
*Urtica dioica*	57	83	47	55	16	21	6	6	7
*Tilia cordata*	52	73	34	35	28	38	10		
*Vaccinium oxycoccos*	51	77	48	66	8	11	5		
*Vaccinium uliginosum*	48	69	46	63	4	6	2		
*Fragaria vesca*	45	62	40	53	5	9	0		
*Hypericum* spp.	43	62	15	15	32	44	4	3	3
*Chenopodium album*	41	41	41	41	0	0	0	1	2
*Quercus robur*	40	66	30	35	19	25	9	5	6
*Plantago major*	39	54	1	1	39	52	1	1	1
*Armoracia rusticana*	37	46	32	39	5	7	0		
*Taraxacum officinale*	33	44	18	19	16	19	1	5	6
*Viburnum opulus*	29	47	18	24	15	23	4		
*Potentilla erecta*	28	44	4	4	25	40	1		
*Rubus caesius*	28	39	28	37	1	2	1		
*Arctium tomentosum*	27	37	1	1	27	36	1		
*Allium ursinum*	22	28	21	27	1	1	0		
*Rosa* spp.	21	33	12	14	11	19	2		
*Pinus sylvestris*	20	43	7	10	15	32	2	1	1
*Syringa vulgaris*	20	21	2	3	18	18	0		
*Acer platanoides*	19	27	16	20	5	6	2	1	1
*Artemisia absinthium*	17	28	1	1	8	10	1	11	17
*Pyrus pyraster*	16	31	11	13	11	16	6	2	2
*Thymus serpyllum*	14	16	8	8	7	8	1		
*Sorbus aucuparia*	14	17	5	6	9	11	0		
*Tanacetum vulgare*	14	18	0	0	14	18	0		

*Abbreviations*: *U* Users across areas, *DUR* Detailed Use Reports across areas, *F_U* Users in Food area, *F_DUR* Detailed Use Reports in Food area, *M_U* Users in Medicinal area, *M_DUR* Detailed Use Reports in Medicinal area, *Overlap F_U/M_U* No of users who used the same taxa in both food and medicinal areas, *V_U* Users in Veterinary area, *V_DUR* Detailed Use Reports in Veterinary area

### Used parts and modes of use

Across all areas fruits and seeds were the most commonly used parts of a plant (36%), followed by leaves (18%), aerial parts (17%), flowers and inflorescences (8%), sap (6%), roots (5%) and twigs (3%), and all other parts (7%). In the food area, fruits alone (without seeds) had an even greater share (48%), as did the use of sap (8%), whereas aerial parts (11%) and flowers with inflorescences (4%) were less important. In the medicinal area, aerial parts and fruits dominated the list, both at 20%, and the proportion of roots (8%), twigs (6%), buds and cones (both 3%), and bark (2%) was also increased compared to their distribution in all three areas combined. The veterinary area is the only area in which the use of leaves had a considerably smaller proportion than across all areas (4%); whereas the use of aerial parts (63%) and bark (6%) had much greater proportions.

The vast majority of use modes across the areas are divided among three possibilities: plants are used either fresh (41%), as a tea or decoction (20%), or dried (12%, including plants dried for recreational tea). In the food area, plants are primarily used fresh (70%), and less often dried (19%), frozen (4%), fermented (4%) or otherwise processed (3%, mainly preserved for winter in the form of compotes, etc.). In the medicinal area, uses are more diverse (about 50 different use modes in contrast to less than 15 in the food area); tea is the most common mode of use (33%), followed by the topical application of fresh plants (13%), tincture (4%), drunk tincture (10%) and decoction (9%). Fresh plants are eaten in only 6% of DUR, while 4% of DUR in the medicinal area refers to whisking in sauna. The eating of dried plants (4%) and jams (3%) as well as making baths (3%, from decoctions) are also quite common. The remarkable presence (in total over 6%) of different food categories (jam, compote, fermented sap, etc.) in the medicinal area stresses the interrelated nature of the two areas. In the veterinary area the primary use modes are decoction (38%) and fresh (15%, mainly fodder), as well as fresh or dried taxa added to fodder (both 12%).

### Past and temporary uses

Seventeen percent of the DUR used in the food and veterinary areas and 10% of the ones from the medicinal area were claimed to have been used only in the past (14% across all areas). Past uses in the food area followed the pattern of general food use, with some exceptions: the most prevalent uses abandoned in the past were snacks and soups (both 25% of all past uses), followed by jams (13%) and recreational teas (5%). Although on a general level past uses were distributed more or less proportionally (one or two uses here and there), some past uses in the food area constituted a considerable number of the uses in specific emic categories: more than half the uses of soup made from *Chenopodium album* and a bit less than half the reports of soup made from *Urtica dioica*. In addition, two-thirds of the uses of the leaves of *Quercus robur* which are put under bread during baking and snacking of the leaves of *Oxalis acetosella* were attributed to the past.

Past uses in the medicinal area are either treatments of childhood diseases (by the mother of interviewees), or uses which respondents had abandoned in later life due to reduced access to the plant (including a decrease of personal mobility) or limited need for healing (for example, cold and some childhood diseases). The majority of past uses are related to general health (mainly prophylactics) and gastrointestinal issues, followed by the dermatological and respiratory categories. The pattern follows quite closely the general distribution of the categories in the medicinal area. Some exceptions, however, exist; for example, the past healing of wounds with leaves from *Acer platanoides* twigs blessed in a church on Pentecost seems to be one of the few remnants of the so-called magical thinking in the quite practical realm of modern domestic medicine in Belarus. A few other ritual practices recalled from the past include: the collecting of *Vaccinium myrtillus* fruits, strictly on St. John’s day, and then drying and storing them apart from other fruits, to be later used to alleviate stomach ache; or wearing dried roots of *Acorus calamus* underneath the clothing for repelling the evil eye. In the veterinary area a few abandoned uses were related to childhood, while others to the past when domestic animals were kept. The majority of past uses, however, in both medicinal and veterinary areas are quite similar to those still used today.

The proportion of uses that were claimed as temporary was relatively small (1% across all areas). However, they also deserve closer attention, as there might be many more of them; a limitation of the method employed in this study is that it did not allow sufficient time for recalling temporary uses. In the food area temporary uses were related either to short-term food shortages in war time (acorns of *Quercus robur* as a bread additive, and fresh leaves of *Urtica dioica* and young cones of *Pinus sylvestris* as snacks), or taste trials (fermentation of *Betula* spp. sap, jam from young cones of *Pinus sylvestris,* salad made from *Taraxacum officinale* leaves and popular jam made from the flowers of *Taraxacum officinale*). Temporary medicinal uses were mostly related to the treatment of young children (the need disappeared when children got older), such as the use of fruits and twigs of *Rubus idaeus* to treat cold and cough, jam of *Taraxacum officinale* against sore throat and bronchitis, fresh fruits of *Viburnum opulus* against cold, leaves of *Tussilago farfara* against cough, aerial parts of *Bidens tripartita* for diathesis, and decoction of aerial parts *Chelidonium majus* to treat chickenpox infection–a use claimed to be prescribed by a doctor. A few uses also related to temporary illness of the interviewee or his or her relatives, such as the use of the leaves of *Vaccinium vitis-idaea* to treat urinary problems or the twigs of *Betula* spp. or *Quercus robur* in a prophylactic bath. One middle aged woman described a ritual for treating her son for epilepsy with the buds of *Betula* spp.: *I went to the crossroad at three o’clock in the morning, gathered buds from nine birches; the next morning I brought nine anthills, covered them with hot water and left them in a hot place for nine days to ferment; I later bathed my son in it*. Given the details of the ritual described, this seems like a historical use tried out in the time of need. However, the woman added that she read about it in a magazine and decided to try it out, and *it helped, he did not perish*. The majority of temporary uses are common in the regional ethnomedicine or official medicine, and in the food area, so in fact those were temporary uses only for specific interviewees.

### Popular attitudes towards the use of wild plants

Quite often, while asking about the use of wild plants, we encountered answers such as: *I know nothing, I am too old, I don’t remember* or *you should ask someone who knows, we don’t deal with such things.* It appeared that people did not consider this kind of knowledge as something worth discussing. After further inquiry, people usually first mentioned forest fruits and all the preserves that they make with them, followed by recreational teas, sometimes trying to change the subject, and also stressing the medicinal properties of the same or other teas (sometimes just knowing the uses, but not really applying the tea for that purpose–and thus not counting as a valid record). Soups and other hot dishes were recalled readily, although younger people seem to be ashamed of knowing how to cook nettle soup, as its use is somehow related with poverty. While condiments constituted a remarkable category, we literally had to name all potential application possibilities (strong alcohol, preservation of cucumbers, etc.), to help people recall their uses. By the end of the interview we often noticed a shift in the attitude towards wild plants and sometimes even the realization of the importance of the subject and interviewees knowledge about it.

Men, talking about the use of wild plants, often referred to women, especially regarding food plants, but in fact there are many quite knowledgeable men as well, even if they do not prepare food or medicate family–they at least know what is done in their family. One of the tasks of men is foraging, especially for tree sap, as well as making home-made alcohol, although officially both activities are illegal. While asking about home-made vodka (and plants soaked in it), we sometimes had to refer to other villagers (without mentioning names)–after hearing that neighbours had taken pride in their moonshine and all it contains, they gladly showed their “harvest”.

Some people remarked that wild plants were useful as long as they were clean, and that now radiation has taken away all their good qualities: *what can you get from a plant after radiation.* Others mentioned that they cultivate all they need, and that earlier (when they were younger) it was easier to just go and collect plants in the wild, but now it is easier to grow them in the garden. Yet some other people said that they never had time to collect in the wild, as work in the collective farm took all their time.

## Discussion

The dominance of Rosaceae and Asteraceae is common in many parts of Europe (see [4] and the references within), and the high number of uses of Ericaceae may be due to the intensive use of forest fruits in both the food and medicinal areas, which may be peculiar to the relatively Nordic location where such taxa grow, seeing that in Estonia the use of Ericaceae is also quite widespread [8, 54]. The low percentage of Lamiaceae, however, is surprizing and may be due to its exclusion from the sample those taxa that were cultivated for food and medicinal purposes. The overlap of the food and medicinal areas (over 55%) is considerably larger than the nearly 40% found in a similar comparison described in [4], which can probably be explained by the absence of cultivated species in this study and the fact that we also included a wider general health category. However, such maximization of the versatility of limited resources is evident, although not emphasized, in many different post-Soviet (or post-socialist) regions (see for example [5, 6, 60]).

While it is not possible to compare the mean number of used plants per person for plants in the medicinal area, the mean number per person of wild plants used in the food area in the region (7.3) was nearly three times lower than the similar value obtained for Saaremaa, Estonia (19.9) [61], but close to the value recorded among Ukrainians living in Romania (7.7) [62]. The FIC for the wild food area (0.94) is, however, slightly higher than the value observed for Estonia (0.91) [61].

The category of salad in the food area deserves specific attention. While not very numerous (only ten taxa), and with a relatively low FIC (0.74), it contains one dominant taxa *Allium ursinum* which is a protected species. Also, the use of *Stellaria media* and *Plantago major* seems to be a remnant from times of food shortage.

Comparison with the only publication providing qualitative data on the recent use of wild food plants in the whole of Belarus [10] shows remarkable overlap within the uses that are numerously represented in this study. There are a considerable number of differences among the seldom mentioned taxa or uses, considering the fact that some of the previously published results were collected from the neighbouring region. This indicates that despite the homogenization of the population during the Soviet era, regional differences may still exist. The high proportion of snacks in [10] and their relatively low occurrence in this study may be attributed to the special emphasis of the questionnaires used by the authors of [10] and the particular interest of their respondents (future botanists) towards nature. However, the reason may also be related to regional cultural differences or, more likely, a considerable decrease of unintended contact with nature, as our interviewees repeatedly stressed that they rarely snacked on anything but berries in the wild.

The temporal division of uses (modest share of past uses, especially within the medicinal area) does not directly support the popular claim that people used more wild plants in the past. However, while folk history is the only applicable method for recalling past uses when there is no historical data available, it is not fully reliable. For example, it is relatively irrelevant to talk about the persistent freezing of wild fruits for preservation purposes, as deep-freezers only became available in the 1980s. However, as this time period is perceived as “long ago” by our interviewees, some of them referred to such a method of preservation as everlasting.

Uses in the medicinal area seem to be more susceptible to literary influence and more diverse in the details of use. For example, inserting the fruits of *Vaccinium oxycoccos* into one’s ear to treat CO-intoxication is one of the few historical medicinal uses of those fruits in Estonia, first recorded in 1984 [63], which most likely originated from one shared literary source. However, while people often acknowledged that they took one or another medicinal uses of wild plants from newspapers or special popular magazines like “Home Doctor”, they also frequently admitted that those or similar remedies were already used by their mothers or grandmothers, so the use of literature is perceived rather as a “refresher” of memory or an “official” acknowledgment of the correctness of the traditional practice (cf. [64]). Nevertheless, some uses are clearly influenced by media, such as the making of jam from *Taraxacum officinale*, which was popularly advertised in the 1980s (cf. [42]). The wide variety of preparation modes and used parts for the most popularly used plants signals either extensive individual experimentation within households or the past presence of a very rich and diverse traditional/regional use of wild plants which has been eroded.

## Conclusions

This study examined the use of wild plants in the food, medicinal and veterinary areas within a small territory limited to one village council in Belarus. It contributes to the documentation of the local use of wild plants and to the understanding of the complex relationship between different use areas of wild plants. We discovered relatively high overlap in the taxa used in the food and medicinal areas and an even higher overlap between the medicinal and veterinary areas. As the majority of taxa with overlapping uses belonged also to the most utilized plants, there appears to be a clear tendency to use plants in several different areas once they are brought into the home. While the number of wild taxa used is relatively high, the mean number of taxa used per person is quite low, which indicates the minor importance of wild plants in the respective areas in the investigated region of Belarus.

## Abbreviations

DUR: Detailed Use Records
FIC: Informant Consensus Factor
UC: Use Citations
